# Method for the extraction of circulating nucleic acids based on MOF reveals cell-free RNA signatures in liver cancer

**DOI:** 10.1093/nsr/nwae022

**Published:** 2024-01-13

**Authors:** Yuqing Sun, Haixin Yu, Shaoqing Han, Ruoxi Ran, Ying Yang, Yongling Tang, Yuhao Wang, Wenhao Zhang, Heng Tang, Boqiao Fu, Boshi Fu, Xiaocheng Weng, Song-Mei Liu, Hexiang Deng, Shuang Peng, Xiang Zhou

**Affiliations:** College of Chemistry and Molecular Sciences, Key Laboratory of Biomedical Polymers-Ministry of Education, Wuhan University, Wuhan 430072, China; Department of Digestive Surgical Oncology, Union Hospital, Tongji Medical College, Huazhong University of Science and Technology, Wuhan 430022, China; Cancer Center, Union Hospital, Tongji Medical College, Huazhong University of Science and Technology, Wuhan 430022, China; College of Chemistry and Molecular Sciences, Key Laboratory of Biomedical Polymers-Ministry of Education, Wuhan University, Wuhan 430072, China; Department of Clinical Laboratory, Center for Gene Diagnosis and Program of Clinical Laboratory, Zhongnan Hospital of Wuhan University, Wuhan 430071, China; Department of Clinical Laboratory, Center for Gene Diagnosis and Program of Clinical Laboratory, Zhongnan Hospital of Wuhan University, Wuhan 430071, China; College of Chemistry and Molecular Sciences, Key Laboratory of Biomedical Polymers-Ministry of Education, Wuhan University, Wuhan 430072, China; College of Chemistry and Molecular Sciences, Key Laboratory of Biomedical Polymers-Ministry of Education, Wuhan University, Wuhan 430072, China; College of Chemistry and Molecular Sciences, Key Laboratory of Biomedical Polymers-Ministry of Education, Wuhan University, Wuhan 430072, China; College of Chemistry and Molecular Sciences, Key Laboratory of Biomedical Polymers-Ministry of Education, Wuhan University, Wuhan 430072, China; College of Chemistry and Materials Science, Hubei Engineering University, Xiaogan 432000, China; Department of Pharmacology, School of Pharmacy, China Medical University, Shenyang 110122, China; College of Chemistry and Molecular Sciences, Key Laboratory of Biomedical Polymers-Ministry of Education, Wuhan University, Wuhan 430072, China; Department of Clinical Laboratory, Center for Gene Diagnosis and Program of Clinical Laboratory, Zhongnan Hospital of Wuhan University, Wuhan 430071, China; College of Chemistry and Molecular Sciences, Key Laboratory of Biomedical Polymers-Ministry of Education, Wuhan University, Wuhan 430072, China; College of Chemistry and Molecular Sciences, Key Laboratory of Biomedical Polymers-Ministry of Education, Wuhan University, Wuhan 430072, China; College of Chemistry and Molecular Sciences, Key Laboratory of Biomedical Polymers-Ministry of Education, Wuhan University, Wuhan 430072, China; Taikang Center for Life and Medical Sciences, Wuhan University, Wuhan 430071, China; Department of Hematology, Zhongnan Hospital of Wuhan University, Wuhan 430071, China

**Keywords:** MOF, circulating cell-free RNA, biomarker, liver cancer diagnosis

## Abstract

Cell-free RNA (cfRNA) allows assessment of health, status, and phenotype of a variety of human organs and is a potential biomarker to non-invasively diagnose numerous diseases. Nevertheless, there is a lack of highly efficient and bias-free cfRNA isolation technologies due to the low abundance and instability of cfRNA. Here, we developed a reproducible and high-efficiency isolation technology for different types of cell-free nucleic acids (containing cfRNA and viral RNA) in serum/plasma based on the inclusion of nucleic acids by metal-organic framework (MOF) materials, which greatly improved the isolation efficiency and was able to preserve RNA integrity compared with the most widely used research kit method. Importantly, the quality of cfRNA extracted by the MOF method is about 10-fold that of the kit method, and the MOF method isolates more than three times as many different RNA types as the kit method. The whole transcriptome mapping characteristics of cfRNA in serum from patients with liver cancer was described and a cfRNA signature with six cfRNAs was identified to diagnose liver cancer with high diagnostic efficiency (area under curve = 0.905 in the independent validation cohort) using this MOF method. Thus, this new MOF isolation technique will advance the field of liquid biopsy, with the potential to diagnose liver cancer.

## Teaser

A breakthrough method for enriching trace circulating nucleic acids in blood using metal-organic framework materials has been proposed, providing a valuable tool for research and early clinical disease diagnosis.

## INTRODUCTION

Cell-free nucleic acids (cfNAs) in human blood, including cell-free DNA (cfDNA) and cell-free RNA (cfRNA), originate from the apoptosis, necrosis, and active secretion of cells from different tissues [1,2]. Therefore, these molecules show the health and status of many solid tissues, with cfNAs having the potential to be used as a ‘liquid biopsy’ for the diagnosis and monitoring of malignant diseases [3–6]. The detection of fetal genomes [7], cancer-associated point mutations [8], methylation [9], and fragmentation patterns [10] of cfDNA in blood have highlighted the utility of cfDNA as a diagnostic or prognostic biomarker tool in the clinic [11]. In contrast to cfDNA, differences in gene expression revealed by cfRNA provide information of numerous disease states; the primary source of circulating cfRNA is active secretion rather than cell death [12] and overexpression of disease-specific transcripts could lead to amplification of disease-derived cfRNA signals in the blood [13]. In this respect, cfRNA represents a window into specific phenotypic information across many tissues and has the potential for application in the clinical diagnosis of various diseases.

cfRNA studies mainly focus on circulating microRNA in blood [14,15]; on the contrary, only few clinical application studies on long cfRNA have been reported focusing, for example, on predicting preterm delivery [16] and early-onset preeclampsia [17], characterization of human bone marrow stimulation and reconstitution [18], screening for Alzheimer disease [19], and cancers [13,20]. The main challenge that limits academic research and clinical application of cfRNA is the lack of a highly efficient cfRNA extraction technology. Despite cfRNA being relatively stable due to associations with proteins and lipoproteins and shielding by extracellular vesicles [4], during the isolation process, the protective layer of RNA is stripped off and cfRNA becomes fragile; therefore, extraction should be fast and preserve RNA integrity [21]. The traditional cfRNA extraction methods are based on silica gel membrane spin columns, whose principle of purification and concentration is based on the interaction between silica gel and nucleic acids, i.e. silanol groups (SiOH) on the surface of the silica gel act as hydrogen donor molecules that can form hydrogen bonds with nucleic acid molecules acting as hydrogen acceptors [22]. The basic principle is that nucleic acids bound to the silica membrane spin column in the presence of a high concentration of chaotropic salt, contaminants are washed away, and the nucleic acid is then eluted from the silica membrane in water or a low-salt buffer. However, the physical and chemical properties of nucleic acid molecules of different chain lengths, as well as DNA and RNA molecules, vary significantly, resulting in a wide range of adsorption and elution efficiencies for DNA and RNA [23]. The length of a double-stranded cfDNA molecule is generally about 170 bp, while single-stranded cfRNA molecules are of many types, such as miRNAs of about 20 nt, and mRNA fragments of several hundred nt in length. The simultaneous purification of DNA and RNA with different fragment lengths in the same system will inevitably lead to inefficient purification of the more complex cfRNA. Furthermore, this approach does not protect cfRNA from degradation. Therefore, there is an urgent need for an efficient, non-biased extraction technique that can effectively preserve the integrity of cfRNA.

In our previous work, we showed that metal–organic framework (MOF) materials have outstanding inclusion capacity for both DNA and RNA [24,25]. Here, we developed a unique method to extract cfDNA, cfRNA, and viral RNA from blood using Co-IRMOF-74-IV (Figs S1 and S2), composed of Co^2+^ and organic linkers (IV), which can adsorb nucleic acids into their pores from blood samples with protein denaturation treatment. This MOF method successfully extracted cfDNA, and content and genetic characteristics were the same as the traditional kit method (QIAamp ccfDNA/RNA Kit, Qiagen, catalog no. 55 184). Importantly, quantity and species richness of cfRNA extracted by the MOF method from the same sample were better than that extracted by the kit method. The quantity of cfRNA extracted by the MOF method was 10-fold better than that extracted by the kit method, and the number of coding RNAs obtained by the MOF method was ∼3-fold that of the kit method, whereas those of long non-coding RNA (lncRNA), small RNA, and pseudogenes and others were ∼10-fold, 4-fold, and more than 10-fold, respectively. Furthermore, the MOF method could enrich the hepatitis B virus (HBV) and hepatitis C virus (HCV) viral RNA in serum, and HBV and HCV copy numbers detected by the MOF method were highly consistent with the routine clinical method (Daan genes^TM^, China, DA-Z070 and Daan genes^TM^, China, DA-Z051). Finally, we conducted a comprehensive characterization of cfRNA in patients with hepatocellular carcinoma (HCC, *n* = 14), chronic hepatitis B (CHB, *n* = 17), and healthy individuals (*n* = 8). Using this data, a model with six cfRNAs (*C1QTNF4, SETBP1, CYBA, PCDHB3, HMGA1*, and *ZNF541*) was developed to diagnose HCC, showing high diagnostic efficiency (area under curve (AUC) = 0.905) in the independent validation cohort (HCC, *n* = 7; CHB, *n* = 4; healthy individuals, *n* = 8), suggesting the huge potential of the MOF method for biomarker identification and biological function research of circulating cfRNA/cfDNA.

## RESULTS AND DISCUSSION

### Extraction process of the MOF method

**Figure 1.**
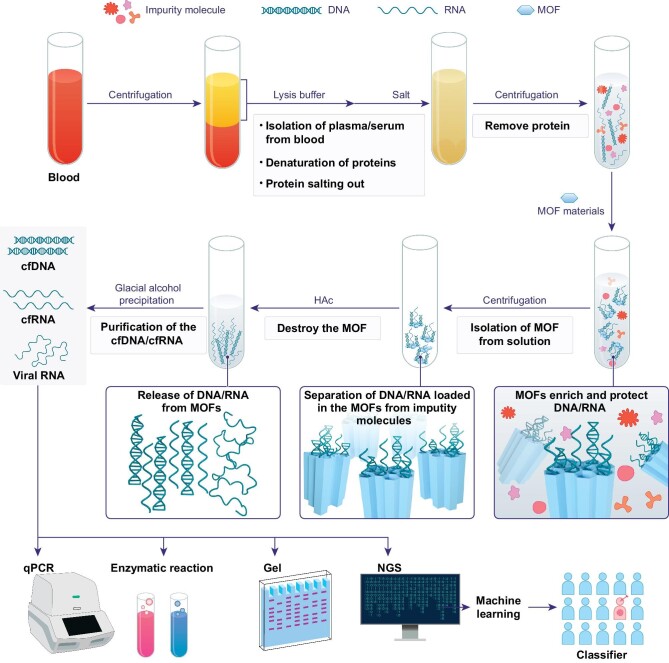
Schematic illustration of the isolation of cfDNA/cfRNA from plasma/serum by the MOF method. NGS: next-generation sequencing.

The process of extracting cfNAs from blood is shown in Fig. 1; first, serum or plasma was obtained from peripheral blood, treated with chaotropic lysis buffer, and salted out to denature and precipitate proteins, leaving all nucleic acids in solution. Subsequently, MOF materials were added to adsorb cfDNA and cfRNA into their pores. Finally, these MOF materials were destroyed to release adsorbed nucleic acids under acidic conditions (2 M acetic acid (HAc), pH = 2.06), followed by purification of nucleic acids with ice-ethanol precipitation. Pure cfRNA was obtained after DNase I treatment, and extracted cfDNA and cfRNA were used to detect mutations/modifications and to quantify gene expression in downstream applications. The procedure is free of phenol and chloroform and requires no prolonged proteolytic digestion steps.

### Evaluation of nucleic acid recovery efficiency and fragment integrity using the MOF method

In order to test the enrichment yield of nucleic acids extracted with the MOF method, nucleic acid fragments of different lengths were added into the serum. The recovery yield of these nucleic acids was then tested and compared with the standard kit method. The recovery efficiency of the MOF method for 33-nt DNA, 22-nt DNA, and 22-nt RNA reached 33%, while the respective recovery efficiencies of the kit method were 27.75%, 0.79%, and 3.07% (Fig. S3). Furthermore, MOF materials were used to enrich total RNA in solution. These MOFs were then opened to release adsorbed total RNA under acidic conditions—the crystalline MOF materials are connected by coordination bonds, nucleic acids can therefore be released by breaking the coordination bonds under acidic conditions (2 M HAc, pH = 2.06). Gel electrophoresis showed that total RNA was released from MOFs with high efficiency and maintained fragment integrity in relation to the total input RNA (Fig. S4). These results prove the high recovery efficiency, universality to different types of nucleic acids, and non-invasiveness of the MOF method.

### Assessment of cfDNA extraction by the MOF method and comparison with a commercial research kit

**Figure 2.**
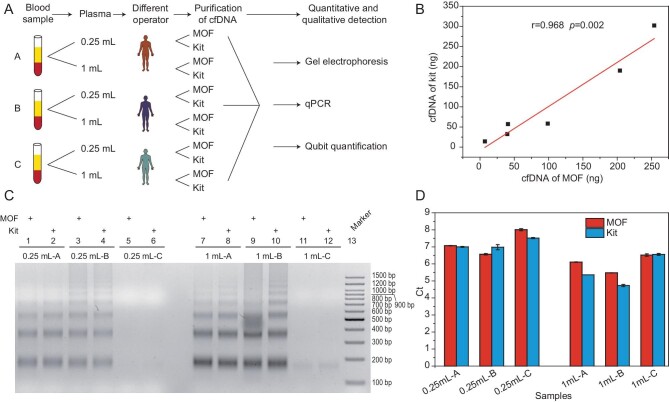
Comparison of the quantity and fragment characterization of cfDNA between MOF and kit methods. (A) Flow chart of comparison with commercial kit. (B) Total quantity of cfDNA tested by Qubit™ 1× dsDNA HS Assay, and the correlation between MOF and kit methods. (C) Comparison of the integrity of cfDNA obtained by the two methods through gel electrophoresis. (D) Detection of the expression of *ALU* in cfDNA through qPCR using MOF and kit methods.

To assess the credibility of the MOF method, extraction efficiency, and the quantity of the extracted cfDNA, a commercial kit method, the most widely used method for extracting cfNA from blood in scientific research (QIAamp), was used as a reference. Three different serum samples from different people (A, B, C) were prepared and cell-free nucleic acids were extracted by different operators from 0.25-mL or 1-mL serum samples using both the MOF method and the kit method, followed by quantitative and qualitative analyses (Fig. 2A). First, the total quantity of cfDNA was highly correlated (r = 0.968, *p* = 0.002) between the two methods using a Qubit™ 1× dsDNA HS Assay Kit (Fig. 2B and Table S1). Additionally, comparison of the integrity of cfDNA through gel electrophoresis revealed the same integrity between the two methods (Fig. 2C). Of note, samples A and B presented the DNA ladder formed from fragmented DNA with integer multiples of 170 bp, and sample C had a lower cfDNA content, concentrated at the position of 170 bp, revealing significant differences between the samples. Therefore, different samples, different operators, and different serum amounts were used to reduce sample, personnel, and sample size errors as much as possible during the comparison process. Additionally, the Ct values of *ALU* gene in cfDNA from these two methods were similar, indicating that the extraction efficiency was comparable (Fig. 2D and Fig. S5). Thus, the extraction efficiency and integrity of cfDNA extracted by the MOF method were highly similar to those of cfDNA extracted by the kit method.

High-throughput sequencing technology was used to evaluate the reproducibility and the difference between the MOF method and the kit method (Fig. S6). Pearson correlation analysis of genes obtained by the MOF method was performed in three parallel repeat experiments (MOF-1, MOF-2, MOF-3), showing identical result of these three repeated experiments on the MOF method (r = 1). Additionally, the Pearson correlation coefficient between the MOF and kit methods was above 0.97 (Fig. S6B), and the length of fragments of both methods were concentrated at 170 bp (Fig. S6C). The copy number variation of cfDNA, integrity, and the content of cfDNA obtained by the MOF method were assessed in repeat experiments with variable aspects and techniques, and compared with the kit method. Thus, the MOF method is stable and reproducible as well as consistent with the kit method with regards to extraction efficiency and cfDNA characterization.

### Extraction of cfRNA by the MOF method and comparison with a commercial research kit

**Figure 3.**
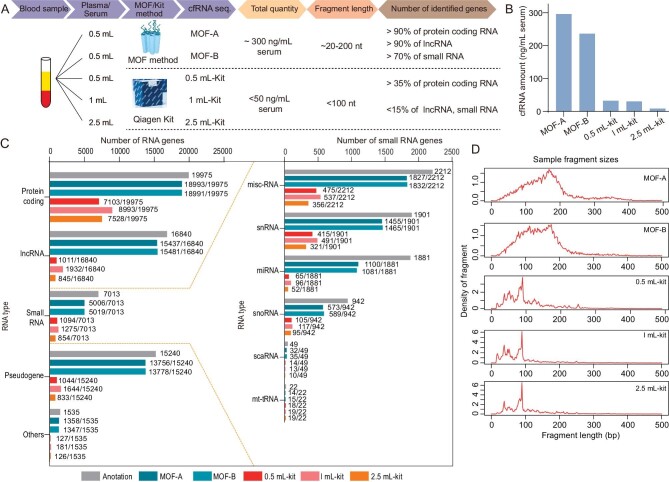
Comparison of the quantity, fragment distribution, and species distribution of cfRNA between MOF and kit methods. (A) Comparison between MOF method and commercial kit method (QIAamp ccfDNA/RNA Kit, Qiagen). (B) Total quantity of cfRNA tested by Qubit™ microRNA Assay Kits. (C) Number of species of RNA detected by MOF and kit methods (FPKM >1). (D) Length distribution of these fragments from MOF and kit methods.

To illustrate the total quantity of cfRNA extracted by the MOF method, pure cfRNA can be obtained after degradation of DNA by DNase I (Fig. S7). The concentration of cfRNA extracted by the MOF method from the same serum sample was ∼300 ng/mL of serum, while the kit method only had 1/10 of the MOF method (Fig. 3B). Furthermore, the expression of cfRNA obtained by both the MOF and kit methods were profiled, a library of the MOF method yielded 5.9 and 5.5 million reads mapping to the human genome, respectively, whereas that of the kit method, with different input serum volume, 0.5 mL, 1 mL, and 2 mL, yielded 3.7, 2.3, and 3.2 million reads mapping to the human genome, respectively. The sequencing data show that for the same amount of input, the sequencing depth of the MOF method is higher than that of the kit method, and the duplication rate is much lower than that of the kit method (Fig. S8). The correlation coefficient of the two replicated experiments using the MOF method was 0.93 and it was above 0.83 for the three replicated experiments using the kit method; however, the correlation coefficient between the MOF method and kit method was lower than 0.5 (Fig. S9A). Thus, the MOF method has high reproducibility, and cfRNA extracted by the MOF method was significantly different with kit methods.

The lengths of RNA fragments enriched by the MOF method were mainly concentrated in the range of 100–200 nt, while those obtained by the kit method were concentrated in the range of less than 100 nt (Fig. 3D), likely because RNA with shorter fragments is preferentially eluted from the column by water, resulting in a greater amount of short fragments enriched by the kit method. Additionally, the pore environment of MOF can protect RNA from enzyme degradation and preserve longer fragments [24].

Additionally, we analyzed the read count distribution of the two methods, showing that reads mainly between 0 and 50 were obtained from the MOF method with a minor amount between 50 and 150. However, the kit method provided less than 25 reads, with the peak closer to 0 than that of the MOF method (Fig. S9B). Thus, the expression of most genes detected by the kit method is lower than that detected by the MOF method, and the latter can detect more low-abundance cfRNA.

We also identified the difference in expression levels of distinct RNA biotypes. GENCODE V.31 annotations downloaded from the GENCODE website (www.gencodegenes.org/) were used to map the expression of distinct RNA biotypes, including protein coding, lncRNA (long non-coding RNA), small RNA (‘misc_RNA’, ‘snRNA’, ‘miRNA’ ‘snoRNA’ ‘scaRNA’ and ‘mt_tRNA’), pseudogene, and others (‘rRNA’ ‘mt_rRNA’ ‘immunoglobulin gene (IG_gene)’ ‘T cell receptor gene (TR_gene)’ and ‘To be experimentally confirmed (TEC)’). Gene expression was set at >1 fragments per kilobase million (FPKM >1), and the number of expressed RNAs of each type that met the condition was counted. A total of 18 993 coding RNAs, 15 437 lncRNAs, 5006 small RNAs, 1358 others, and 13 756 pseudogenes were identified by MOF-A, corresponding to 95.1%, 91.7%, 71.4%, 88.5%, and 90.3% of annotated transcripts of each respective RNA biotype. The same results were observed in the biological replicate (MOF-B), with 95.1% of coding RNAs, 91.9% of lncRNAs, 71.6% of small RNAs, 87.8% of others, and 90.4% of pseudogenes being identified. Consistent results from repeated experiments demonstrated robust reproducibility of the MOF method. Contrastingly, we identified a total of 7103 coding RNAs, 1011 lncRNAs, 1094 small RNAs, 127 others, and 1044 pseudogenes with the 0.5 mL-kit, corresponding to 35.6%, 6.0%, 15.6%, 8.3%, and 6.9% of annotated transcripts of each respective RNA biotype. Similar results were observed with the 1 mL-kit and 2.5 mL-kit (45.0%, 11.5%, 18.2%, 11.8%, and 10.8% for the 1 mL-kit; 37.7%, 5.0%, 12.2%, 8.2%, and 5.5% for the 2.5 mL-kit), showing that the number of expressed coding RNAs, lncRNAs, small RNAs, pseudogenes, and others obtained by the MOF method is about 3-fold, 10-fold, 4-fold, and >10-fold those obtained with the kit method (Fig. 3C). Furthermore, the number of various types of small RNAs obtained by the MOF method reached more than 60% of the annotated amount, and the number of ‘misc_RNA’ and ‘snRNA’ was 3-fold that of the kit method, about 11-fold in ‘miRNA’ and 5-fold in ‘snoRNA’. Thus, the most abundant cfRNAs obtained by the MOF method are protein coding RNAs and lncRNAs, which can reach more than 90% of the annotation amount. On average, the overall RNA obtained by MOF was above 3-fold that obtained by the kit, of which lncRNAs and miRNA reach up to 10-fold that of the kit method. Thus, the RNA species obtained by the MOF method are more abundant, more information on RNA can be retrieved, and low-abundance RNA can be detected. Additionally, the proportion of lncRNA detected by the MOF method reached ∼20%, while that detected by the kit method was less than 4% (Fig. S9C), indicating that the proportion of lncRNA obtained by the kit was very small and biased. Additionally, to illustrate the authenticity of the results, the same comparison experiment was conducted in another batch of plasma samples. cfRNA extracted by the MOF method had a higher quantity (Fig. S10), high reproducibility (Fig. S11), longer fragment length (Figs S12 and S13), and more RNA types (Fig. S14) compared with the kit method.

We compared the MOF method with silica gel membrane spin column-based kits for cfDNA/cfRNA extraction, and found that there was little difference in cfDNA extraction efficiency. The MOF method was even more efficient than the kit method for cfRNA extraction. We speculate that there are two reasons for this. First, our previous work reported that MOF materials adsorb nucleic acids into the pores of its interior, protecting nucleic acids from nuclease degradation in serum [24]. Therefore, RNA extracted by the MOF method is much higher than that of traditional kit extraction methods based on silica membrane adsorption. DNA is much more stable than RNA, so when comparing the MOF method with the kit method, the difference is not obvious. Second, silica gel membrane spin column-based kits can result in differences in the adsorption and elution efficiencies of DNA and RNA. The structure and properties of DNA and RNA molecules vary significantly, resulting in a wide range of adsorption and elution efficiencies for DNA and RNA [22,23]. The length of a double-stranded cfDNA molecule is generally about 170 bp, while single-stranded cfRNA molecules are of many types, such as miRNAs of about 20 nt and mRNA fragments of several hundred nt in length. The simultaneous purification of DNA and RNA with different fragment lengths in the same system will inevitably lead to inefficient purification of the more complex cfRNA. In addition, we compared our method with the magnetic bead-based approach, and the results showed that the magnetic bead method cannot simultaneously extract both cfDNA and cfRNA (Fig. S15).

### RT-qPCR of serum HCV RNA by the MOF method and comparison with the clinical standard

**Figure 4.**
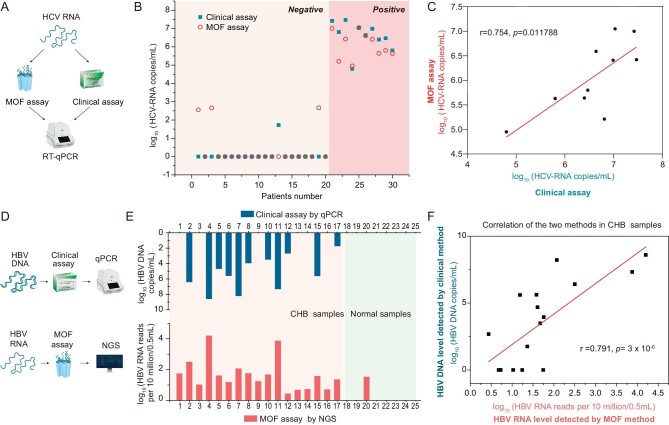
Comparison of the ability of the MOF assays and clinical assays to detect virus levels in serum. (A) Schematics of HCV RNA detection by MOF and clinical assays. (B) HCV copy numbers detected by clinical assays (Daan genes^TM^, China, DA-Z070) and MOF assays. (C) Correlation of HCV copy numbers between these two assays in 10 samples with both HCV positive diagnoses. (D) Schematics of HBV DNA and RNA detection by clinical assays and MOF assays, respectively. (E) HBV DNA copy numbers in serum detected through Taqman qPCR assay using clinical assays (Daan genes^TM^, China, DA-Z051), HBV RNA level detected by transcriptome sequencing using MOF assays, and their correlation (F).

RT-qPCR of HCV RNA in serum was conducted on samples extracted by the MOF method and compared with results using the clinical kit method (Fig. 4A). HCV RNA copy numbers were detected in 30 serum samples (Fig. 4B and Table S2); the kappa statistical test indicated that the two methods had high consistency (Kappa = 0.724, *p* = 0.000063, Table S3). The correlation of HCV copy number between the two methods in 10 samples with both positive diagnoses (r = 0.754, *p* = 0.011788) was extremely high (Fig. 4C), showing that HCV copy number detection is highly consistent in both the MOF method and the clinical detection method. Thus, the MOF method could extract virus RNA from blood with high accuracy and good reproducibility. Furthermore, given the linear working curve, the MOF extraction method has high accuracy and stability (Fig. S16).

### HBV RNA copy number detection using MOF-sequencing and comparison with clinical detection methods

HBV RNA was analyzed in samples from a patient with CHB and in healthy individuals through cfRNA transcriptome sequencing (Fig. 4D). Reads aligned to the HBV genome were counted, gene expression of HBV RNA was normalized in RPM (reads per 10 million mapped reads), and log_10_ (RPM, HBV RNA reads per 10 million mapped reads) was used for the evaluation of HBV and compared with the clinical method detection parameter log_10_ (HBV DNA copies) (Table S4). The number of HBV RNA reads was undetectable in samples from healthy individuals, while an obvious HBV RNA signal was observed in samples from patients with CHB (Fig. 4E). Additionally, the correlation coefficient r of HBV expression level between the MOF and clinical detection method was 0.791 (*p* < 0.001) (Fig. 4F), consistent with the correlation coefficient reported in the literature [26], indicating that serum HBV RNA and HBV DNA had a good correlation (Pearson correlation ∼0.7). Thus, the signal detected by our MOF method was as accurate as the clinical method and proved that the MOF extraction method is suitable for downstream detection of large-scale high-throughput sequencing.

### Sources of cfRNA

To identify the tissue of origin of cfRNA, a tissue deconvolution analysis of cfRNA was performed in samples from healthy individuals (Fig. S17). The result shows that half of cfRNAs were present in blood circulation originating from blood cells, with significant contributions from transcripts expressed in the lung, liver, and kidney, all of which are parenchymal organs with a rich blood supply and important functions in blood metabolism. This is similar to the report of cfDNA origin in plasma, where blood cells are the main contributors to the plasma cfDNA pool, followed by placenta (in pregnant females) and liver [27]. Thus, this indicated that cfRNA from blood contains transcripts specifically expressed in liver tissues, yet overshadowed by transcripts from blood cells.

Through gene set enrichment analysis [28], we investigated the overall biological functions of the differentially expressed cfRNAs between HCCs and healthy controls. The upregulated cfRNAs were most significantly enriched in activation of pathways in cancer (Fig. S18). The increased cfRNA in HCC samples may not originate from tumor tissue alone but are nevertheless involved in tumor progression pathways. Therefore, cfRNA in serum contains liver cancer-specific cfRNA and has potential as an HCC diagnostic biomarker.

### Identification of HCC-specific cfRNA biomarker candidates and evaluation of their potential diagnostic value for HCC

**Figure 5.**
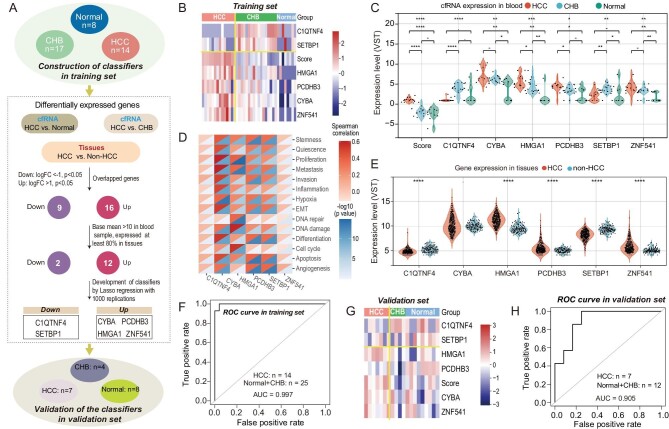
Identification of HCC-specific cfRNA biomarker candidates and evaluation of the diagnostic efficacy of the model. (A) Flow chart of the identification process of cfRNA biomarker candidates. (B) Distribution of expression levels of the six cfRNA candidates in HCC, CHB, and healthy groups of the training set. (C) Expression levels of these six cfRNA candidates in the training set (HCC, CHB and healthy groups) from plasma samples. (D) Correlation analysis of the expression of the six genes with 14 cancer hallmark pathways in HCC tissues (TCGA-LIHC). (E) Expression levels of the six candidate genes in tissues from TARGET GTEx dataset. (F) AUROC of the cfRNA signature to discriminate HCC from CHB and healthy tissue samples together in the training set. (G) Expression distribution and (H) AUROC of model biomarkers in an independent validation cohort including HCC, CHB, and healthy tissue samples.

CHB causes almost 40% of HCC, which is the second leading cause of cancer-related mortality worldwide [29]. Therefore, it is particularly important to monitor patients with CHB for progression to liver cancer. Despite advances in monitoring HBV-related HCC, there are currently no reliable biomarkers that accurately predict the onset of HCC in the setting of HBV infection. Therefore, samples from patients with CHB and from healthy individuals were used to identify HCC-specific diagnostic biomarkers (Fig. 5A). Here, we collected serum samples from 14 patients with HCC, 17 patients with CHB, and eight healthy individuals from Zhongnan Hospital as a training set (population characteristics are listed in Table S5; inclusion and exclusion criteria for all groups are shown in first part of Section 9, Supplementary Materials). cfRNA extracted from 0.5 mL of serum obtained from the above 39 serum samples was sequenced; approximately 10 ng of cfRNA was used to generate each sequencing library.

#### Development of the cfRNA biomarkers for HCC diagnosis

A total of 114 downregulated and 559 upregulated cfRNAs were identified in HCC and healthy tissue samples, and 6878 downregulated and 293 upregulated cfRNAs were identified in HCC and CHB samples (Fig. S19). In the TCGA TARGET GTEx dataset, 5151 genes were downregulated and 4339 genes upregulated between HCC tumor tissues and non-tumor tissues (Fig. S19). In order to obtain liver tissue-derived cfRNAs, we collected overlap of the above differentially expressed genes between serum samples and tissue samples, resulting in nine downregulated genes and 16 upregulated genes (Fig. S20). Subsequently, we limited the basal expression levels (cfRNA base mean >10 and expressed in at least 80% of samples) for cfRNAs in blood and mRNAs in tissues, respectively, and obtained two downregulated genes and 12 upregulated genes after excluding some low expression genes. cfRNA expression levels in these three groups of the 14 genes (Fig. S21) showed significant expression differences between HCC and non-HCC samples. Of note, *H19* and *RP5-1171I10.5* are lncRNAs and the remaining 12 genes were all protein coding RNAs. The emergence of lncRNA is due to the comprehensive and efficient cfRNA extraction capability of the MOF method, which can obtain more than 90% of the lncRNA, but the kit method can only obtain a small amount of cfRNA, thus losing many potential biomarkers.

To generate a robust diagnostic model for HCC, the least absolute shrinkage and selection operator (LASSO) regression analysis with 1000 iterations [30] was applied to screen cfRNA candidates with diagnostic value for HCC, and the coefficients obtained by LASSO regression were used as weights to generate a cfRNA score for HCC diagnosis. The combination of *C1QTNF4, SETBP1, CYBA, PCDHB3, HMGA1, ZNF541*, and *GBP2* (rank by frequency) appeared most frequently by LASSO regression (Fig. S22). However, the diagnostic performance of the seven cfRNAs mentioned above was only mildly improved compared with the first six cfRNAs, and greatly improved compared with the first five cfRNAs. Therefore, our cfRNA-score was generated based on the first six cfRNAs, that was, cfRNA-score = (–0.65310) × C1QTNF4 + 0.15879 × CYBA + 0.27373 × HMGA1+ 0.29307 × PCDHB3 + (–0.21822) × SETBP1 + 0.12482 × ZNF541 + (–2.25578). These six cfRNA signatures and cfRNA score showed the capability to discriminate HCC from CHB and healthy tissue samples (Fig. 5B, C). Correspondingly, the differentially expressed signature of these six genes was also significantly observed in HCC tissues compared with healthy tissues (Fig. 5E). However, subtle differences in the gene expression pattern between blood and tissues can be observed in Fig. 5C and E. There are maybe two main reasons: one reason is that half of cfRNAs were present in blood circulation originating from blood cells, while only a small portion comes from the liver. Thus, these six genes may not only originate from the liver, and contributions from other tissues cannot be ruled out. The second reason is that the pattern of entry of RNA from the cells into the bloodstream is not clear. It is possible that not all RNA from liver tumor cells enters the bloodstream, but does so selectively. Therefore, there are subtle differences in the expression profiles of these six genes in the blood and in the tissues.

The performance of the 6-cfRNA signature for HCC diagnosis was assessed using the area under the receiver operating characteristic curve (AUROCs), with AUROCs for the distinction of HCC from CHB and healthy tissues together being 0.997 (Fig. 5F) and from CHB and healthy tissue separately being 0.996 and 1.00, respectively (Fig. S23).

#### Validation of cfRNA biomarkers in a separate cohort

The performance of the 6-cfRNA signature for HCC diagnosis was then validated in an independent cohort with 19 serum samples (HCC, *n* = 7; CHB, *n* = 4; healthy, *n* = 8; population characteristics listed in Table S6), showing that the model effectively segregated HCC and control samples (CHB and healthy tissue samples) with good diagnostic efficiency (AUROC = 0.905; Fig. 5G, H). Thus, cfRNA alterations can be used to non-invasively diagnose patients with HCC.

### Biological function study of the six cfRNA biomarkers

To explore the function of these genes in HCC, we calculated the correlation between gene expression in HCC tissues (TCGA-LIHC) and signals of 14 cancer hallmark pathways from a database of cancer SEA (30 329 142) (Fig. 5D). *CYBA* was found to be associated with most functional states in cancer cells, especially proliferation, metastasis, and differentiation. *HMGA1* was closely related to DNA damage and cell cycle in HCC, which are recognized as critical factors in tumor development and progression [31,32]. Additionally, we conducted a literature search on the function of these genes and their association with liver disease (Table S7), showing that only the *HMGA1* and *CYBA* genes were associated with liver cancer progression, and studies on the correlation of other genes with the progression of liver cancer were largely absent. *HMGA1* promotes tumor growth and migration in HCC [33–35], and *CYBA* is related to hepatitis and liver fibrosis [36,37]. There are very few studies investigating the relationship between *PCDHB3* and *ZNF541* genes and cancer progression, and there is only one article reporting that *PCDHB3* is related with colorectal cancer and ovarian cancer [38]. For the *C1QTNF4* gene, the pathological relevance with tumorigenesis and cancer-related inflammation are not well documented. Li *et al.* postulate that CTRP4 (C1qTNF-related protein 4) may be a potential inflammatory cytokine in cancer cells, suggesting that C1qTNF4 related protein is a novel tumor-promoting inflammatory regulator [39]. For the *SETBP1* gene, Kohyanagi *et al.* reported that SE translocation (SET) is a cancer-promoting factor whose expression is upregulated in many cancers. *SETBP1* mutations have also been identified in solid tumors including pancreatic neuro-endocrine tumors, breast cancer, non-small cell lung cancer, and gastric cancer [40]. However, Li *et al.* reported that downregulation of *SETBP1* promoted non-small cell lung cancer progression by inducing cellular EMT and disordered immune status [41].

We investigated the expression of these two genes, *C1QTNF4* and *SETBP1*, in various tumor tissues, and found that these two genes were down-expressed in many tissues, and the down-expressed *SETBP1* could be clearly observed in HCC tissues as compared with non-HCC tissues (Fig. S24). The expression of the gene *C1QTNF4* was very low in HCC tissues (Fig. S25). In HCC, the role of *C1QTNF4* in prognostic deterioration or prognostic protection is unclear (Fig. S26). Although some reports have claimed that genes *C1QTNF4* and *SETBP1* are cancer-promoting factors in tumors [39,40], there are very few relevant studies and essentially none in HCC. Therefore, the biology and functions of these genes in the blood circulation system and tumor lesions, especially in HCC tissues, need further investigation and verification.

## CONCLUSION

Progress in the development of cfRNA-based applications is slow despite strong early interest. The biological function and clinical diagnosis of cfRNA need further exploration, which urgently requires technological breakthroughs in related fields, including (1) efficient cfRNA extraction technology, (2) various types of cfRNA library construction methods, and (3) accurately identify specific sets of diagnostic biomarkers from a large pool of cfRNAs during data processing. More importantly, standardizing the cfRNA detection process, specifying sample collection and storage conditions, cfRNA extraction methods, and library construction methods, and developing data analysis processes will greatly promote the clinical application of cfRNA. Herein, we propose a novel and efficient MOF-based cfRNA extraction method with the advantages of high extraction efficiency and preservation of RNA integrity compared to the most widely adopted kit method. Our results reveal the high reproducibility and excellent extraction capability of the MOF method, suggesting that this method has great potential for technical support in cfRNA functional and clinical research. We preliminarily demonstrate that cfRNA contains information for the diagnosis of liver cancer and identify potential diagnostic biomarkers using this MOF method; nevertheless, further cohort samples are needed to validate the results.

## EXPERIMENTAL METHODS

### Cell free nuclei acids extraction by MOF enrichment method

Refer to Part 3 of Supporting information—Circulating nuclei acids extraction by MOF enrichment method. Blood collection was approved by the Ethics Committee of Zhongnan Hospital, Wuhan University (Approval Number: 2017058).

### cfRNA library construction

cfRNA library was constructed by SMARTer Stranded Total RNA-Seq Kit—Pico Input Mammalian (Takara Bio USA, Inc., 635005), and the operation steps were performed according to the instructions provided by the manufacturer.

### HCV RNA quantitative analysis by the MOF method

HCV RNA positive quantitative reference sample (Daan genes^TM^, China, DA-Z070) was used. HCV RNA concentrations are 1.0 × 10^3^, 1.0 × 10^4^, 1.0 × 10^5^, and 1.0 × 10^6^ IU/mL, respectively. These were used for quantitative detection of the HCV RNA concentration in the clinical plasma. RNA was extracted from 200 μL HCV RNA positive quantitative reference plasma sample, clinical blood from HCV-infected patients and none-HCV-infected individuals by our MOF enrichment method, respectively. And these RNAs were used as a template to operate the RT-qPCR, and the data of the HCV RNA positive quantitative reference plasma sample was used to calculate the linear relationship between the quantification cycle (Ct) values.

### Identification of HCC-specific cfRNA candidates and evaluation of the diagnostic efficacy of the model

#### Differential expression analysis

We used R package ‘DESeq2’ to conduct differential expression analysis. We used |logFC|<1 and *p* < 0.05 as cutoff for significance.

#### Normalization of cfRNA expression data

We normalized cfRNA expression data before performing downstream analysis. We used R package ‘DESeq2’ to calculate a variance stabilizing transformation (VST) from the fitted dispersion-mean relations and then transformed the count data (normalized by division by the size factors or normalization factors), yielding a matrix of values which are now approximately homoskedastic (having constant variance along the range of mean values) [42].

#### LASSO regression

We used R package ‘glmnet’ to conduct LASSO regression to screen cfRNA markers for HCC diagnosis (20808728). Penalty parameter lambda was set to ‘lambda.1se’ by default. Type measure parameter was set to ‘AUC’ for diagnosis study. Cross validation was carried out by ‘3-fold’. The LASSO regression analysis was repeated by 1000 times to count the selection frequency of cfRNA markers and marker combinations.

#### GSEA for the differentially expressed cfRNAs

Differential expression analysis between 14 HCCs and eight controls was conducted using R package ‘DESeq2’. Input genes for GSEA were sorted by their logFC values. Signaling pathways activated or suppressed by the input genes were decided by the NES value derived from GSEA using R package ‘clusterProfiler’ [43]. We used p.adjust <0.25 for significance.

#### GSVA for the key genes

ssGSEA was used to calculate separate enrichment scores for each pairing of HCC sample and gene sets of 14 cancer hallmark pathways (http://biocc.hrbmu.edu.cn/CancerSEA/) by R package ‘GSVA’ [44]. The ssGSEA score was further rescaled by the min-max normalization method. Correlation analysis was performed between expression values of key gene and NES of signaling pathways.

#### Deconvolution analysis

The signature matrix for deconvolution analysis was derived using TCGA TARGET GTEx dataset according to Larson's method [45]. CIBERSORTx (https://cibersortx.stanford.edu/) was used to impute gene expression profiles and provide an estimation of the abundances of member cell types using cfRNA expression data [46].

#### Statistical analysis and visualization

Microsoft R Open v4.0.2 was used for data mining, bioinformatics analysis and visualization in transcriptomics data.

## DATA AND MATERIALS AVAILABILITY

The data that support this study are available from the corresponding authors upon reasonable request. The sequencing data of cfDNA and cfRNA generated in this study have been deposited into the Gene Expression Omnibus (GEO) under the accession number GSE210775. All other data needed to evaluate the conclusions in the paper are present in the paper and/or the Supporting information.

## Supplementary Material

nwae022_Supplemental_File
