# Supplementary material for: Method for the extraction of circulating nucleic acids based on MOF reveals cell-free RNA signatures in liver cancer
Source: Natl Sci Rev. 2024 Jan 13;11(1):nwae022. doi: 10.1093/nsr/nwae022 (PMC10860518; doi:10.1093/nsr/nwae022)
Supplement: nwae022_Supplemental_File [file nwae022_supplemental_file.pdf]

Supporting Information for

Method for the Extraction of Circulating Nucleic Acids based on MOF Reveals Cell-Free RNA Signatures in Liver Cancer

Yuqing Sun[1†], Haixin Yu[3,4†], Shaoqing Han[1†], Ruoxi Ran[2], Ying Yang[2], Yongling Tang[1], Yuhao Wang[1], Wenhao Zhang[1], Heng Tang[1], Boqiao Fu[7], Boshi Fu[8], Xiaocheng Weng[1], Song-Mei Liu[2*], Hexiang Deng[1*] and Shuang Peng[1*], Xiang Zhou[1,5,6*]

Corresponding author: Song-Mei Liu, smliu@whu.edu.cn; Hexiang Deng, *hdeng@whu.edu.cn;* Shuang Peng, pengshuang@whu.edu.cn*;* Xiang Zhou, *xzhou@whu.edu.cn*

1. Blood sample preparation

Blood samples were collected in VACUETTE K3 EDTA tubes for plasma processing or Vacutainer tubes for serum processing. Blood samples were kept at room temperature and samples were processed within 2 h after blood draw. The blood was centrifuged (10 min, 1,500g, 4 ℃); the supernatant was transferred to fresh 1-2 mL tubes and centrifuged again (10 min, 3,000g, 4 ℃); and the supernatant was used as plasma or serum for follow-up experiments. The plasma or serum was used fresh or flash frozen and stored at -80 ℃ for long-term storage. Freeze/thaw cycles were avoided.

2. Commercial kit for circulating nuclei acids extraction

QIAamp ccfDNA/RNA Kit (QIAGEN, Germany)

The most widely used commercial method on the scientific research for extracting cfNA from blood is a product developed by Qiagen company (QIAamp ccfDNA/RNA Kit, QIAGEN, Germany, catalog no. 55184). The extraction technique is based on the physical properties of nucleic acids, the solubility of nucleic acid is reduced in the ethanol environment, and it is adsorbed to the surface of the column material in the form of precipitate, and is separated from other impurity molecules (proteins, salts, etc.). Then, the nucleic acid is eluted from the column material with water to achieve the effect of purification and enrichment, which is a common way of purifying and enriching nucleic acid. However, this method also has some problems. There are certain differences in the physical properties of DNA and RNA, the purification and recovery efficiency of DNA and RNA are different, and the purification and recovery efficiency of nucleic acids with different fragment lengths are different, so this purification method exists nucleic acids type and fragment length preference.

3. Circulating nuclei acids extraction by MOF enrichment method

3.1 cfDNA/cfRNA extraction through MOF enrichment method

The extraction step as follows:

(1) Take 500 μL of plasma sample into 1 mL EP tube, add Guanidine thiocyanate solution to lyse serum/plasma, vortex for 5 seconds, and after mixing, let it stand at room temperature for 3 minutes.

(2) Add $ZnCl_2$ solution and vortex for 1 min immediately, milky white insoluble matter appears, place on ice for 3 min.

(3) Centrifuge at 12000g for 5 min, milky white precipitate can be seen. The supernatant is a clear and light yellow liquid. Transfer the supernatant to a clean EP tube for later use. 500 μL of plasma can get 500 μL of supernatant. Add 6 μL Tris (1M, pH=10.89), mix well, and centrifuge briefly.

(4) MOF material adsorbs cfDNA: Add 60 μL of 10 mg/ml IRMOF-74-IV material to the solution obtained in step (3), and react the above reaction on a rotating instrument at room temperature for 2 hours.

(5) After the reaction is over, centrifuge the above reaction at 12000g for 15 minutes, remove the supernatant, and collect the precipitate (the precipitate is a pink solid).

(6) Destroy the MOF material to release cfDNA: Add 20 uL of 2 M Hac (pH 2.06) to the pellet, mix by pipetting for 1 min, and leave it at room temperature for 5 min. It can be seen that the pink solid disappears and white flocculent insoluble matter appears.

(7) Purification of step (6) to obtain cfDNA/cfRNA:

Ice ethanol precipitation: (a) Add 80 uL of water to the mixture obtained in step (7), and then add 10 μL of sodium acetate (3 mol/L, pH=5.2) and 1 μL glycogen, mix well. (b) Add 300 μL of pre-chilled ice ethanol, mix well, and place it at -80 ℃ for 30 minutes to 2 hours or overnight; (c) Centrifuge at 12000 g for 20 minutes, carefully remove the supernatant, and aspirate all the droplets on the tube wall; (d) Add 75% ice ethanol with 1/2 of the centrifuge tube capacity, pipette three times, centrifuge at 12000g for 10 minutes, carefully remove the supernatant, and aspirate all the droplets on the tube wall; (e) Place the uncapped EP tube on the laboratory table for 5 minutes at room temperature to evaporate the remaining liquid to dryness; (f) Add 20 μL of

RNase-free water to dissolve the cfDNA/cfRNA solid at the bottom of the EP tube, and place it in a refrigerator at -20 ℃ for later use or -80 ℃ for long-term storage. We obtain the circulating cell-free nucleic acids, including cfDNA and cfRNA, this solution was used in downstream experiments of cfDNA.

3.2 Purification of cfRNA

Add 1uL DNase I and 2 uL DNase I buffer into the solution (obtained from part 3.1 step 7), react at 37 ℃ for 1h, and then purify the cfRNA by RNA purification kit, RNA Clean & Concentrator™-5 (ZYMO RESEARCH, R1013, USA), the operation steps were performed according to the instructions provided by the manufacturer.

3.3 *Characterization of the MOF material*

The material used in this work is the same as that reported in our previous work, in which we have fully characterized the material, including PXRD, N2 adsorption analysis, stability of the MOF materials in various buffer conditions, etc. In this project, we also performed PXRD characterization for each synthesized material, and the characterization results are shown in Figure S1. The PXRD data of Co-IRMOF-74-IV were collected on a Rigaku Smartlab 9 kW diffractometer operated at 45 kV, 200 mA for Cu Kα (λ = 1.5406 Å). The good agreement between the pattern of activated Co-IRMOF-74-IV and that of the simulated model indicated the material has the same crystal structure as the simulated one.

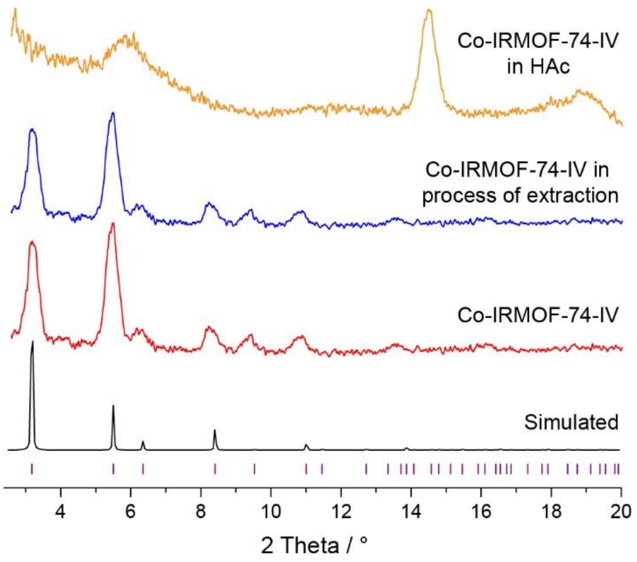

Figure S1. PXRD patterns of Co-IRMOF-74-IV samples were tested in various conditions on a Rigaku Smartlab 9 kW diffractometer operated at 45 kV, 200 mA for Cu Kα (λ = 1.5406 Å). Red line: Activated Co-IRMOF-74-IV without treatment. Blue line: Co-IRMOF-74-IV adsorbed cfNA after incubation with lysis plasma for 2 h. Yellow line: Adsorbed cfNA Co-IRMOF-74-IV treated with HAc for 5 min.

At the same time, we used SEM to characterize the material morphology, see Figure S2. The SEM images of Co-IRMOF-74-IV were collected on an FEI Verios-460 with accelerating voltage of 500.0 V，the samples were prepared by sprinkling directly onto the sample stages without coating. All the Co-IRMOF-74-IV samples exhibit rod shapes that are micrometer in length with uniform sizes.

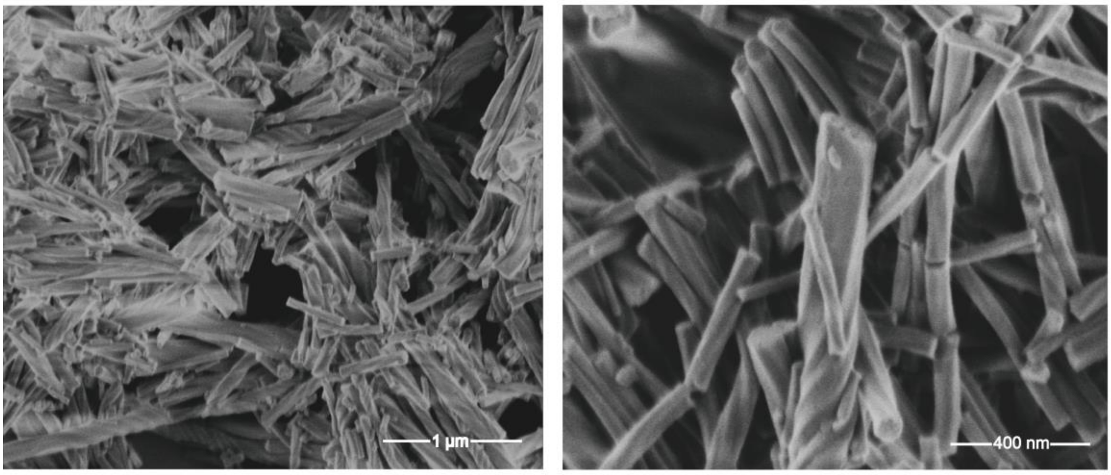

Figure S2. SEM images of Co-IRMOF-74-IV, scale bar, 1 μm (a) and 400 nm (b).

3.4. Stability of the MOF materials throughout the extraction-elution process and mechanisms of MOF nucleic acids extraction

First, we tested the PXRD of the MOF material and the whole extraction process. The material was incubated with lysed blood for 2h, and then centrifuged to obtain the MOF material adsorbed with circulating nucleic acids (cfNA). The peak patterns of the PXRD spectrum (Figure S1, blue line) are identical to those of the untreated material (Figure S1, red line). This indicates that the crystal structure of MOF material is the same as that of the untreated material during the adsorption of cfNA,

and the material is stable during the adsorption of cfNA. The next process is the elution of cfNA from the MOF material. We took advantage of the unstable nature of MOF material under acidic conditions and used HAc to destroy the structure of MOF material to release free nucleic acids from the material. Therefore, we tested the stability of the material after incubation with HAc (2 M, pH=2.06), and the PXRD results (Figure S1, yellow line) showed that the crystal structure of the material was all destroyed after the addition of HAc. In summary, MOF is stable and maintains its crystal structure during the adsorption of free nucleic acids from lysed plasma. However, when HAc was added to destroy the structure of MOF during the elution of cfNA, the crystal structure of the material was completely destroyed, so that the cfNA could be released.

Our previous work has demonstrated that Co-IRMOF-74-IV has a high adsorption capacity for DNA/RNA and adsorbs nucleic acids to the inside of the MOF pore [*Nat. Commun*., **2018**, 9(1): 1293; *J. Am. Chem. Soc.* **2020**,142, 5049-5059], so we apply this capacity to adsorb very low amounts of circulating nucleic acids in the blood. These very low amounts of nucleic acids can be used as markers for disease diagnosis, but because of their extremely low levels in the blood, silica gel column and magnetic bead methods are not efficient for their extraction. Based on the fact that the Co-IRMOF-74-IV material is unstable under acidic conditions, the circulating nucleic acids were released from the MOF material by destabilizing the structure of the MOF material using HAc (2 M, pH=2.06). Finally, the released nucleic acids were purified by ice-ethanol precipitation and used for downstream analytical experiments, the nucleic acid extraction process is shown in Figure 1 in the main text.

4. **Valuation of recovery efficiency and fragment integrity used MOF method**

Recovery efficiency of short DNA/RNA addition in the plasma

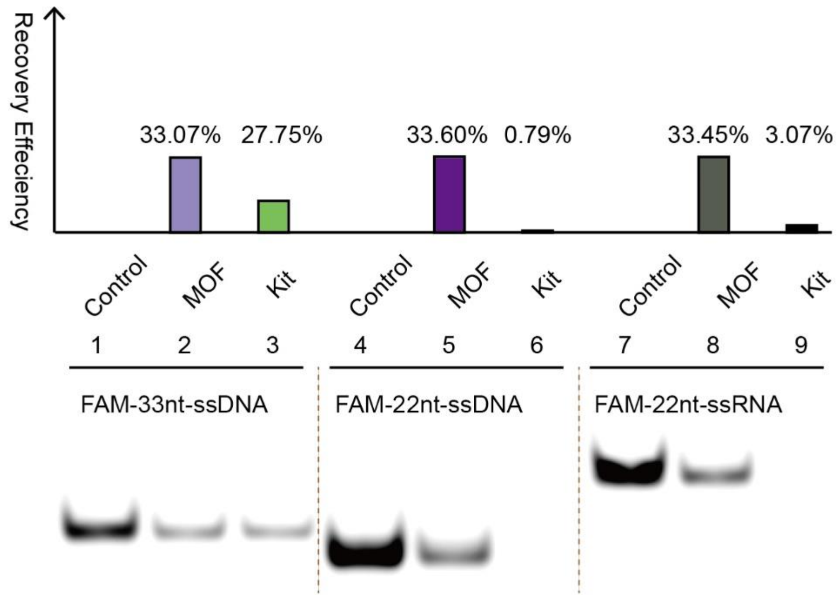

Figure S3. Comparation recovery yield of different lengths oligonucleotide added into plasma using MOF method and kit method through polyacrylamide gel electrophoresis, sequence used in this experiment is list in Table S8.

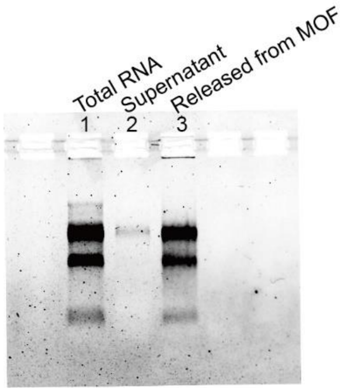

Figure S4. Assessment of total RNA released efficiency and fragment integrity using MOF method.

5. cfDNA detection

At first a lot of plasma from different people was mix up, then cfDNA was extracted from 250 μL and 1000 μL plasma by MOF enrichment method and commercial kit, respectively. Finally, all cfDNA was dissolved in 20 μL H_2O, which was used as the template for qPCR detection and dsDNA Qubit quantification. These assays were performed with three biological repeats by three different laboratory

staffs.

5.1 qPCR for cfDNA detection and comparation

All qPCR reactions were performed as 10 μL reactions using 2 × Hieff qPCR SYBR Green Master Mix (Yeasen, 11201) and amplified on a CFX-96^{TM} Real-Time System (Bio-Rad, USA). The qPCR primers for ALU115 sequence used in this study were forward: 5‘-CCTGAGGTCAGGAGTTCGAG-3’ and reverse: 5’-CCCGAGTAGCTGGGATTACA-3’ (Sangon biotech, China), are listed in Table S8 (ALU115-F stands for forward primer; ALU115-R stands for reverse primer). Reactions of 10 μL contained 5 μL of Hieff qPCR SYBR Green Master Mix; 1 μL of 10 μmol/L ALU115-F; 1 μL of 10 μmol/L ALU115-R; and 2 μL of template cfDNA. Thermal cycling was performed on a CFX-96^{TM} Real-Time System (Bio-Rad, USA) under the following conditions: 95 ℃, 5 minutes; 95 ℃, 20 seconds; 55 cycles of 95 ℃ for 3 seconds, 60 ℃ for 30 seconds. Fluorescence was measured at 60 ℃ for each cycle (*1*). All assays were performed with at least three technical replicates.

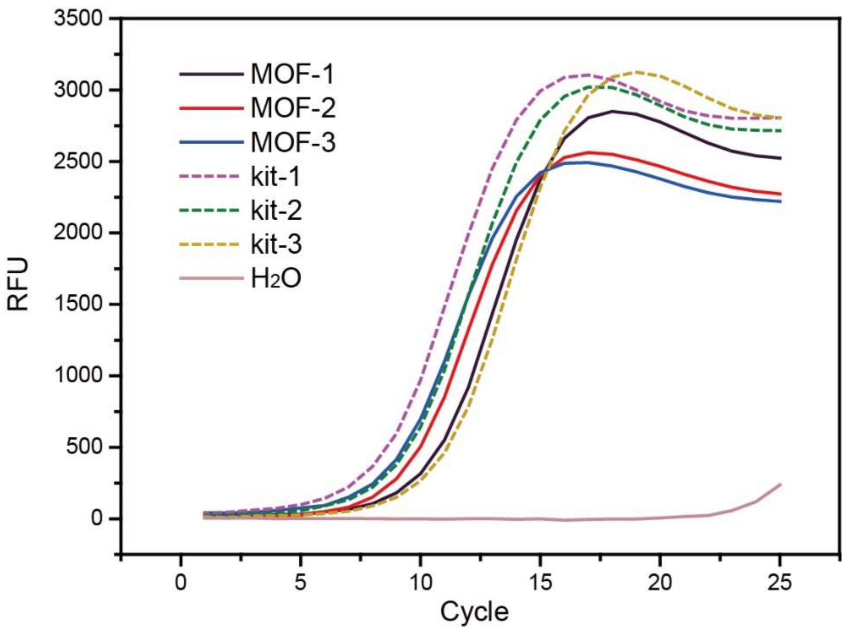

Figure S5. Detection the expression of ALU gene in cfDNA through qPCR using MOF and kit method (repeat the experiment three times).

5.2 cfDNA quantitively detection by Qubit kit

The concentration of cfDNA was test by Qubit™ 1X dsDNA HS Assay Kits (Invitrogen™, Q33230), and Qubit™ Flex Fluorometer, the operation steps were performed according to the instructions provided by the manufacturer.

Table S1. Total quantity of cfDNA was tested by Qubit™ 1X dsDNA HS Assay

	Quantity of cfDNA (ng)			
	0.25 mL plasma		1 mL plasma	
	MOF	Kit	MOF	Kit
A	40.8	57.2	204	190
B	98.8	58.4	254	302
C	7.12	14.16	39.8	32.4

5.3 NGS analysis of cfDNA

cfDNA library construction

cfDNA library construction through NEBNext Ultra II DNA Library Prep Kit (NEB, E7645S), the solution from part 3.1 step 7 directly used as input DNA without fragment.

Analyses of WGS data from cfDNA

Whole-genome NGS data for cfDNA samples with 150 base-pair pair-end reads from Illumina sequencing were first sent for the adaptor and quality trimming using cutadapt (*2*). Reads shorter than 25 nt after trimming were excluded. Processed reads were then mapped to the human genome (hg38) by Bowtie2 with the default parameters. Coverage of different samples were calculated for each 100 kb bin, the correlation between samples was calculated by plotCorrelation in deepTools 2.0.

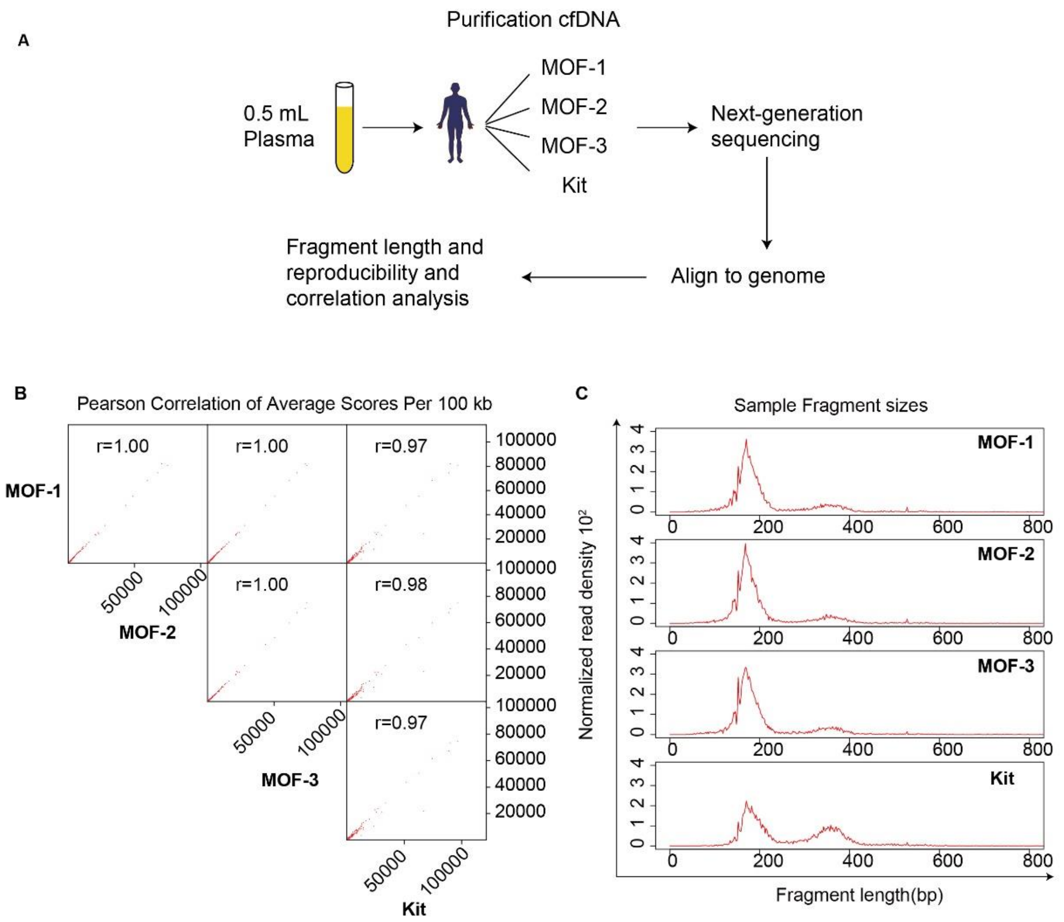

Figure S6. **Comparation the fragment characterization and correlation of cfDNA between MOF and kit methods through sequencing technology.** (A) Evaluation the stability of the MOF method and discrimination from kit method using High-throughput sequencing. (B) The correlation of cfDNA expression level (MOF-1, MOF-2, MOF-3) obtained by the MOF method in three parallel experiments and kit method. (C) The length distribution of these fragments.

6. cfRNA analysis

cfRNA quantitively detection by Qubit kit:

The concentration of cfRNA was test by Qubit™ microRNA Assay Kits (Invitrogen™, Q32880), and Qubit™ Flex Fluorometer, the operation steps were performed according to the instructions provided by the manufacturer.

cfRNA library construction:

cfRNA library constructed by SMARTer Stranded Total RNA-Seq Kit - Pico Input Mammalian (Takara Bio USA, Inc., 635005), the operation steps were performed according to the instructions provided by the manufacturer.

Quantification of cfRNA fragments

The human hg38 genome and list of transcripts v31 were downloaded from Gencode (www.gencodegenes.org). Raw FASTQ reads were trimmed to remove adaptor contamination using cutadapt. We include a 10 bp random barcode (NNNNNNNNNN) ligated to the fragments during library construction, random barcode serves to identify PCR duplicates from real different fragments with the identical sequences. After removing the random sequence, clean reads were aligned to the human reference genome using STAR 2.7.5a (*3*). Only the proper pair and uniquely mapped alignments was persisted for the downstream pipelines. Feature Counts v2.0.1 was used to count reads on gene, the number of different types of RNA were counted based on the annotation file from Gencode.

6.1 Comparison of the quantity cfRNA between MOF method and kit method (Qiagen).

To illustrate that DNase I degraded all of the cfDNA completely, we introduced 400 nt-length dsDNA to show the efficiency of DNase I degradation. We added 400 nt-dsDNA to a mixture of cfDNA and cfRNA extracted from plasma, then added DNase I and reacted at 37 ℃ for 1 h, and finally performed electrophoresis experiments by 1% agarose gel electrophoresis of the reaction products. As shown in Figure S7, the electrophoretic bands of 400nt-dsDNA and cfDNA disappeared after incubation with DNase I, indicating that both 400nt-dsDNA and cfDNA were completely degraded by DNase I, and there was no interference of DNA in the system. cfRNA has molecules of various lengths and low content, and gel electrophoresis was performed using gel red dye for staining display, which is less effective in imaging single-stranded RNA, and therefore no bands of cfRNA can be seen in the gel

electrophoresis pictures. The above steps for degradation of 400 nt-dsDNA and cfNA are consistent with the steps for obtaining cfRNA in the main text, indicating that DNase I degradation of DNA is still very complete and pure cfRNA can be obtained.

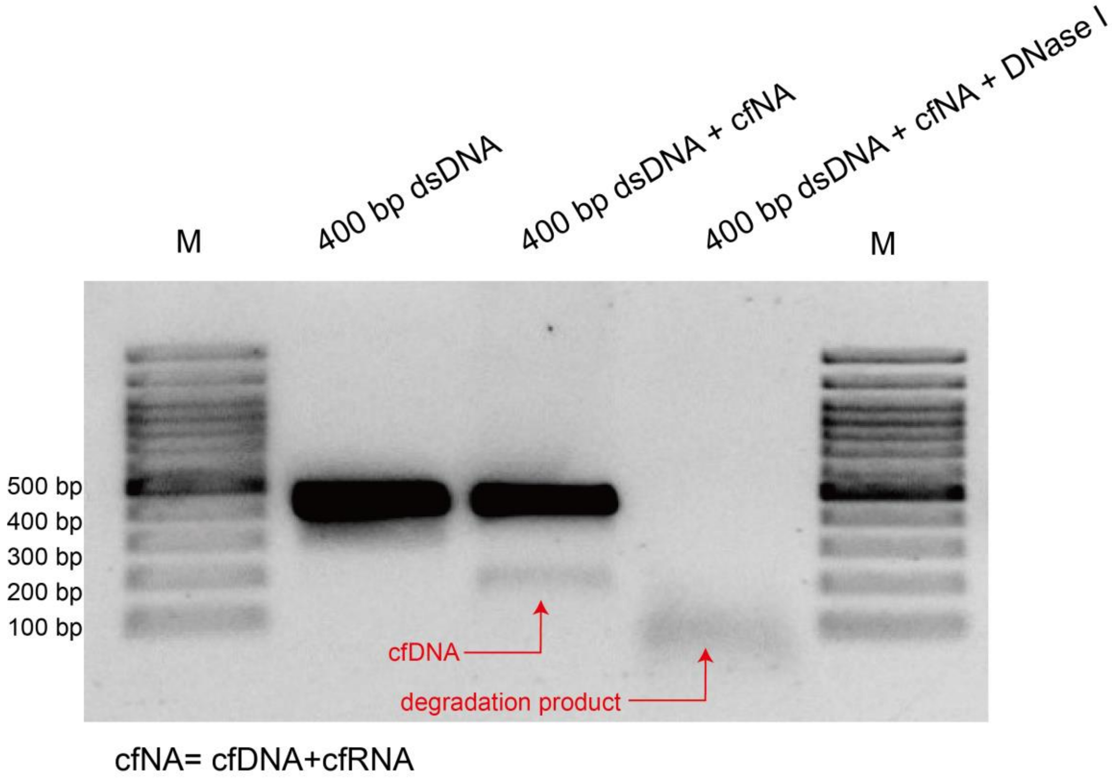

Figure S7. 1% agarose gel electrophoresis of the reaction products of cfDNA and 400nt-dsDNA mixture after DNase I treatment.

The quality of sequencing is closely related to the quantity of input RNA during library construction, so when we compare the two methods by sequencing, the quantity of input is the same (calculated according to the microRNA qubit determination method). According to the reviewer's comment, we recalculated the cfRNA comparison data of the two methods in the main text to get the sequencing depth and the duplication rate data. These new results were now added to the revised supplementary information (Figure S9). The data show that for the same amount of input, the sequencing depth of the MOF method is higher than that of the kit method, and the duplication rate is much lower than that of the kit method. In other words, the kit method yields a low number of cfRNA species and results in libraries with low

complexity. This new detailed information about the library preparation of these two methods also confirms the better RNA diversity obtained by the MOF method.

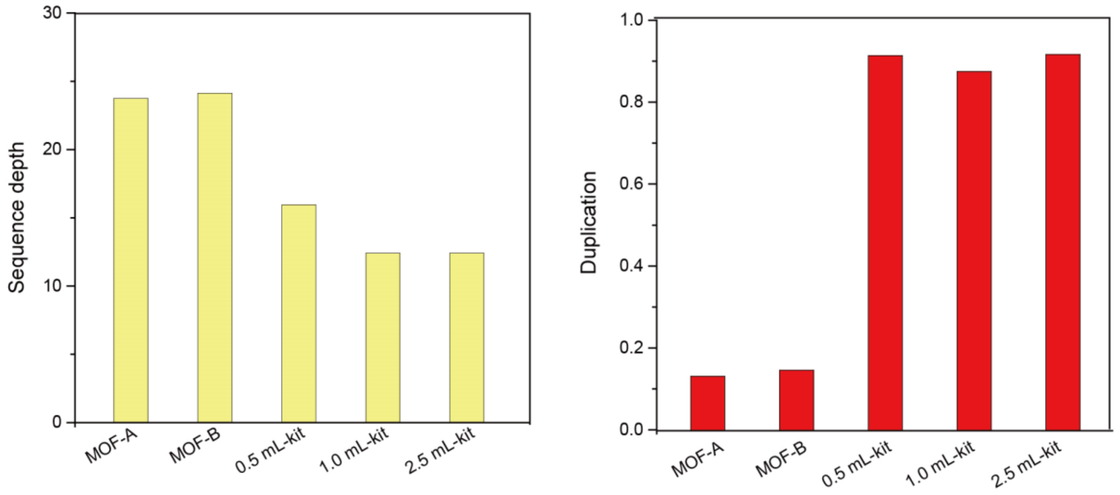

Figure S8. Sequencing depth and the duplication rate of cfRNA library using MOF method and kit method, respectively.

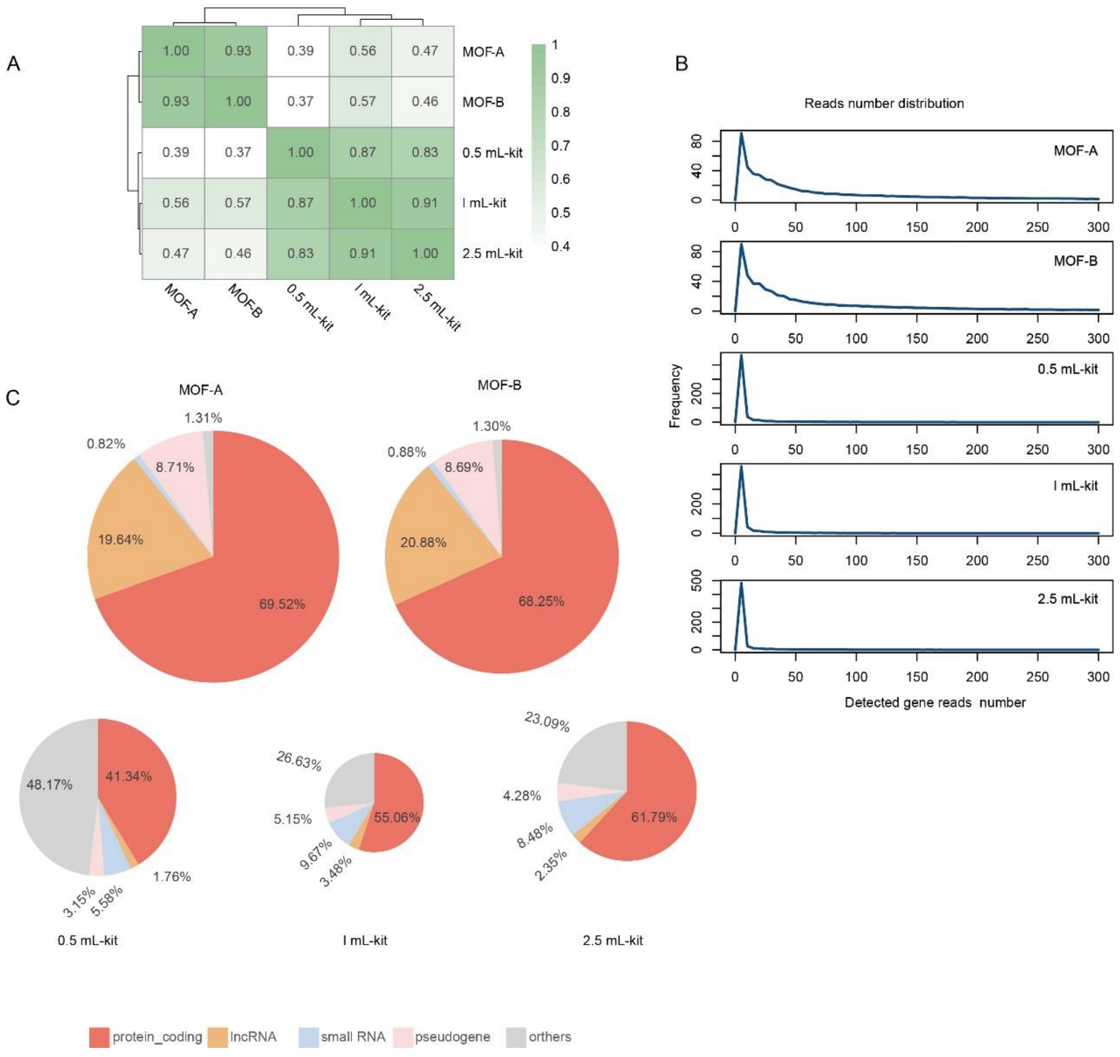

Figure S9. Comparation the quality, fragment distribution, and species distribution of cfRNA between MOF and kit methods. (A) The correlation of cfRNA expression level obtained by the MOF method in two parallel experiments (MOF-A, MOF-B) and kit method (different input serum volume, 0.5 mL,1mL and 2 mL). (B) The reads count of different types of RNA species in MOF and kit method. (C) Proportion distribution of different types of species RNA in MOF and kit method.

In order to increase the accuracy of the comparison results of these two methods, we repeated the analysis of cfRNA content, fragment length, and gene type in another batch of plasma samples, and the results are as follows:

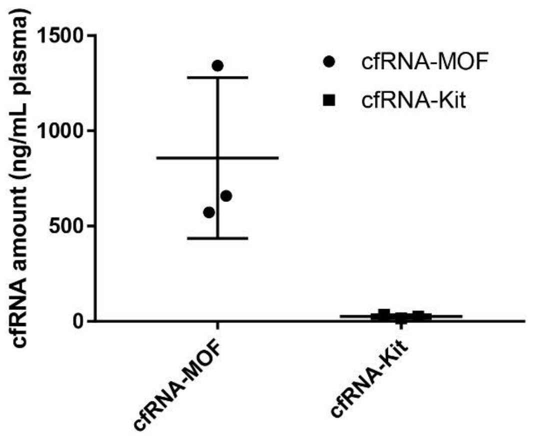

Figure. S10. Total quantity of cfRNA was tested by Qubit™ microRNA Assay Kits used by MOF and kit methods.

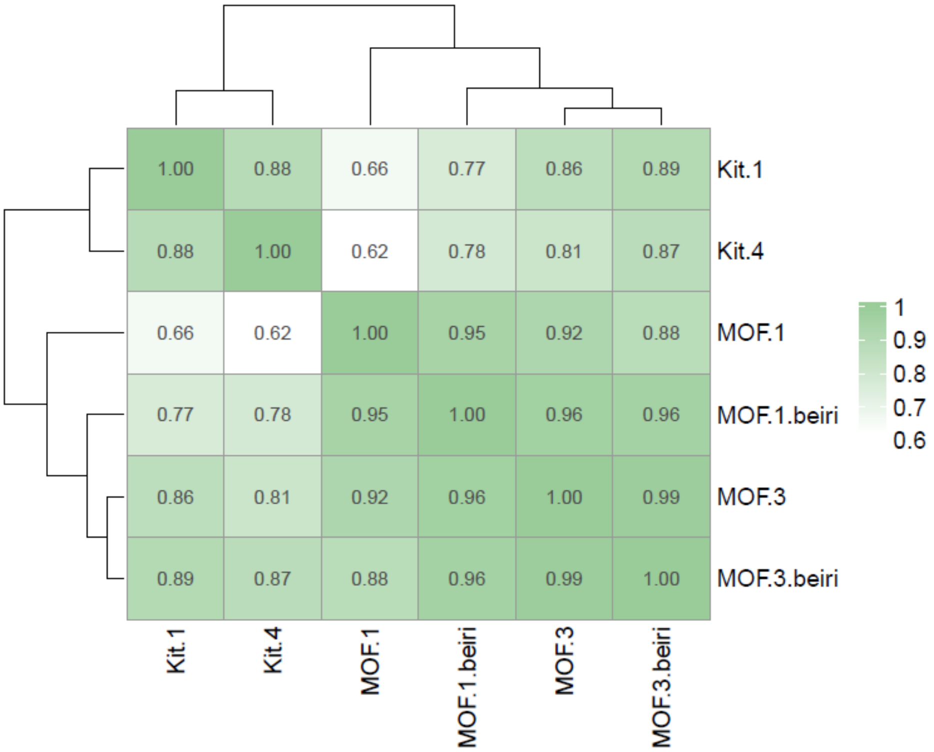

Figure S11. The correlation of cfRNA expression level obtained by the MOF method and kit method from 0.5 mL plasma in two parallel experiments, including MOF method (MOF-1, MOF-3) and kit method (kit-1, kit-3).

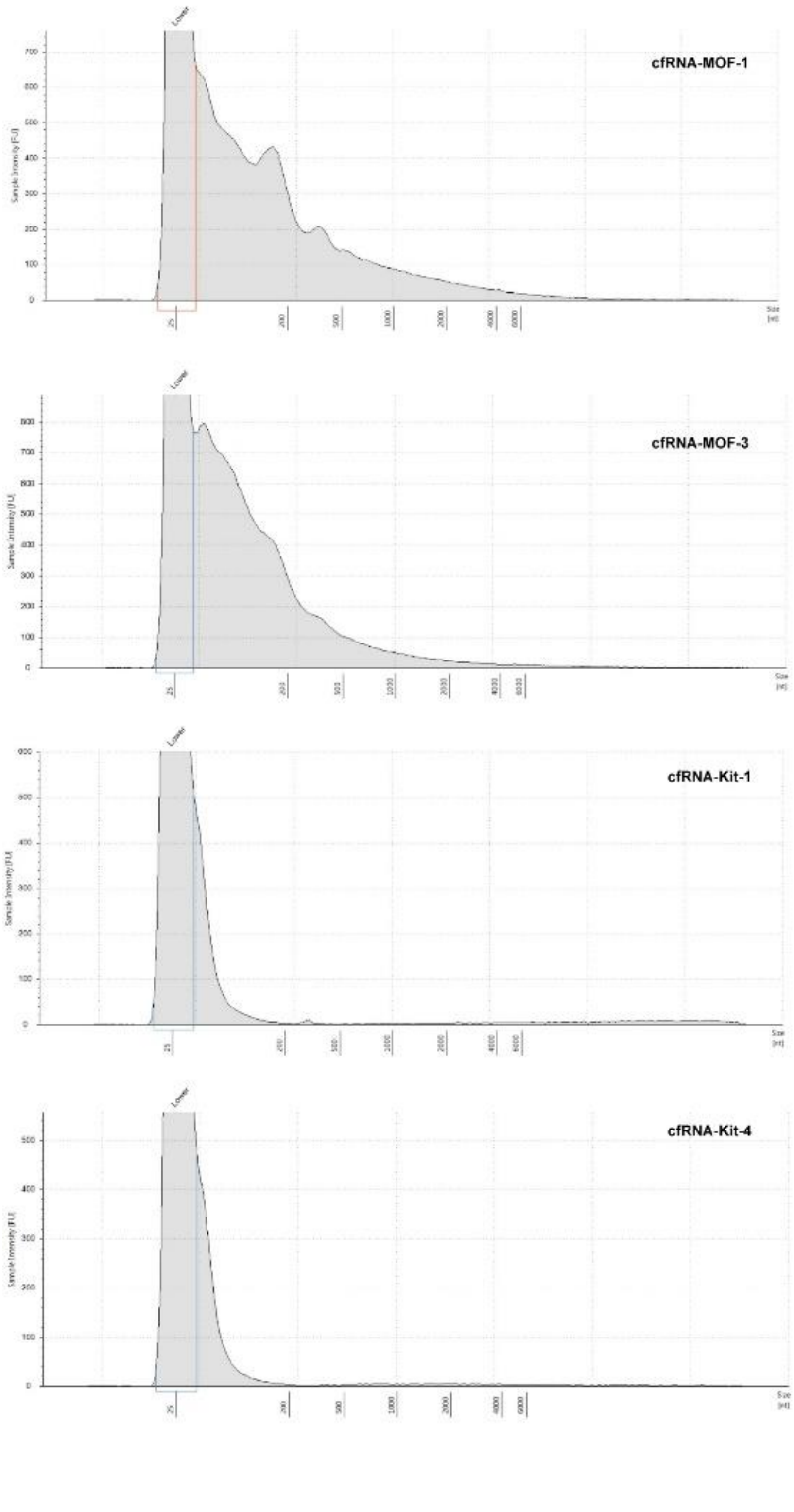
cfRNA-MOF-1
cfRNA-MOF-3
cfRNA-Kit-1
cfRNA-Kit-4

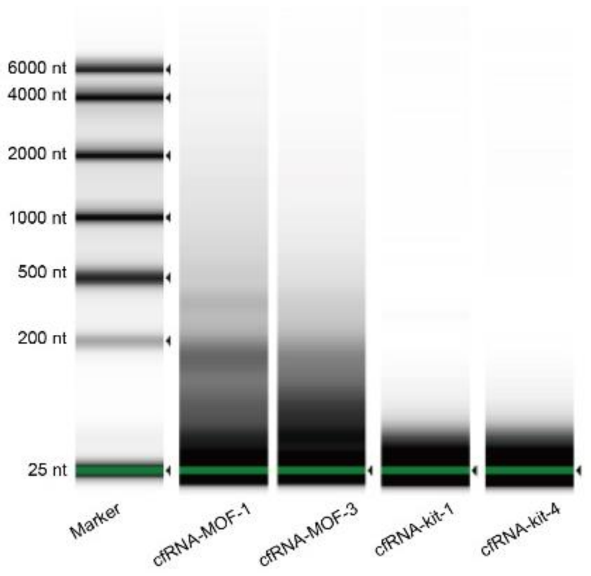

Figure S12. cfRNA fragments length distribution was detected by the Agilent 2100 Bioanalyzer, and was calculated through sequencing data.

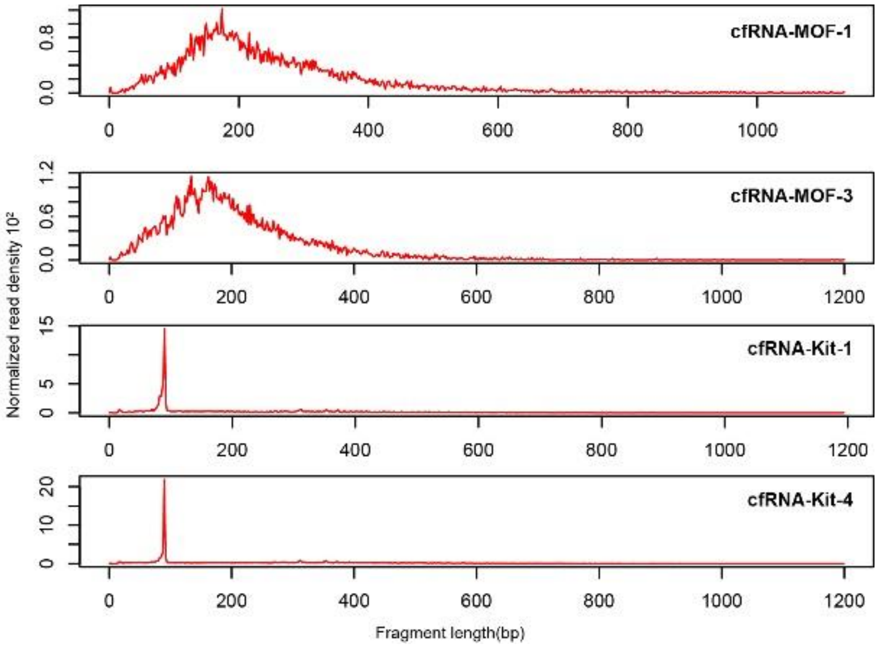

Figure S13. cfRNA fragments length distribution was calculated through sequencing data.

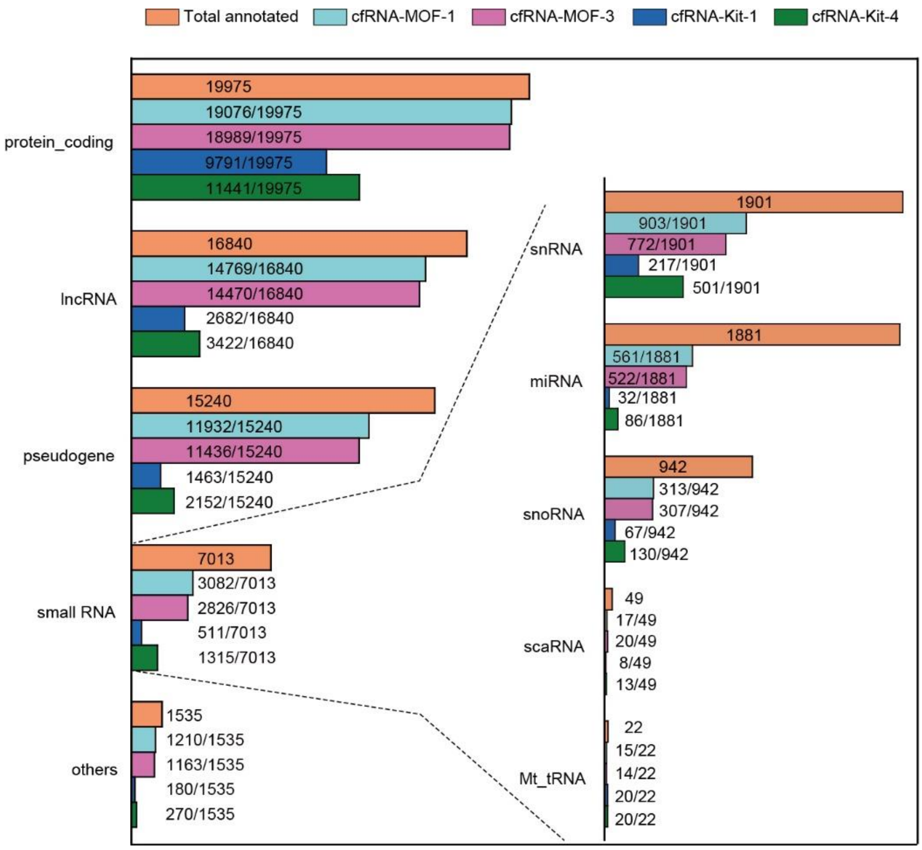

Figure S14. Number of different kinds of species RNA detected in MOF and kit method (fpkm>1)

6.2 Comparison of the quantity of cfDNA and cfRNA between MOF method and magnetic bead method (VAHTS).

We investigated and found two products for magnetic bead purification of nucleic acids, one is MagMAX™-96 Blood RNA Isolation Kit (Invitrogen™, Catalog Number AM1837), and the other is VAHTS Serum/Plasma Circulating DNA Kit (Vazyme™, Catalog Number N902). Since the instruction manual of the first kit states that it is only suitable for RNA extraction from whole blood, which is inconsistent with the target of our extraction, we chose the VAHTS Serum/Plasma Circulating DNA Kit (VAHTS) to compare with our MOF method.

The same mixed plasma sample was extracted by MOF method and VAHTS, and then the amount of cfDNA and cfRNA was detected separately. The results are shown

in Figure S15, the amount of cfDNA extracted by VAHTS is 1.18 times higher than that of MOF method (Figure S15A). The same result can be observed in the data of qPCR assay again (Figure S15B-C). For the cfRNA comparison results (Figure S15D-F), the VAHTS method was not effective in extracting 18S rRNA of cfRNA from plasma (Ct values were the same as those of the control group without template). Such a result was expected because the operation manual of the VAHTS method states that it is only suitable for cfDNA extraction. We hypothesize that this is because the buffer conditions for the magnetic beads to adsorb and elute DNA and RNA are not the same, and therefore RNA and DNA cannot be extracted at the same time.

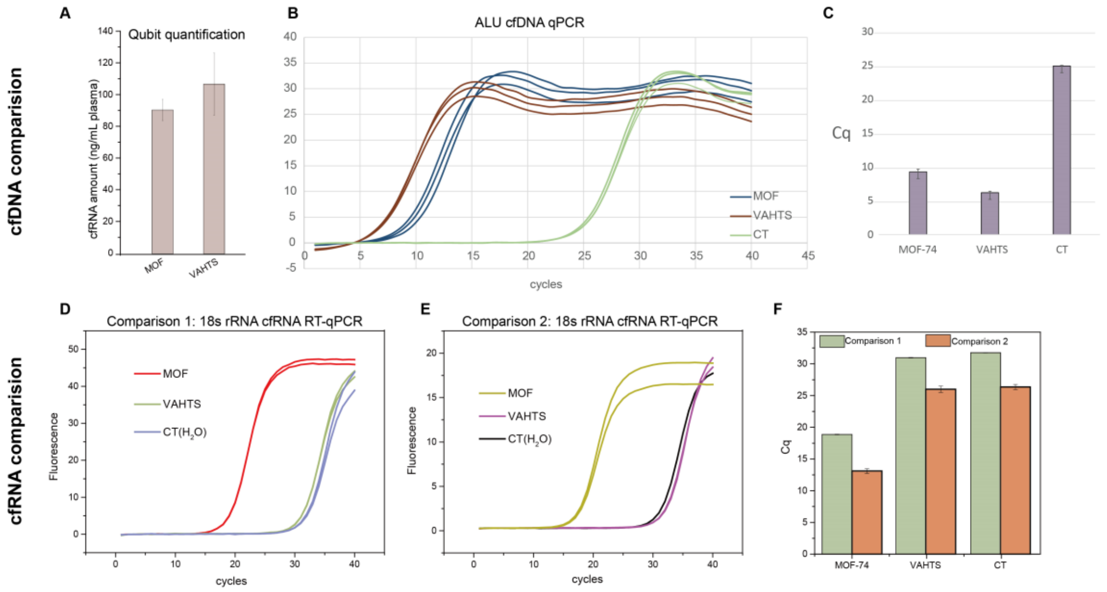

Figure S15. Comparison of the quantity of cfDNA and cfRNA between MOF method and magnetic bead method (VAHTS). A) qubit quantification of cfDNA. B) and C) ALU cfDNA quantification by qPCR. D-F) 18s rRNA of cfRNA quantification by RT-qPCR.

7. RT-qPCR for HCV RNA detection in clinical serum samples

Reactions of 20 μL contained 5μL of TaqMan™ Fast Virus 1-Step Master Mix (Thermo Fisher Scientific Inc., 4444432); 0.5 μL of 10 mmol/L forward primer F (Sangon biotech, China); 0.5 μL of 10 mmol/L reverse primer R (Sangon biotech,

China); 0.5 μL of 10 mmol/L HCV Taqman probe (Sangon biotech, China), which was labeled with the fluorophore carboxyfluorescein at the 5’ end (5’FAM) and quencher minor groove-binding at the 3’ end (3’MGB); and 2 μL of template RNA (RNA was extracted from 250 μL serum and dissolved in 20 μL H_2O), these experimental design was referred reported work (*4*) and these primer and probe are list in Table S8. Thermal cycling was performed on a CFX-96™ Real-Time System (Bio-Rad, USA) under the following conditions: 50 ℃, 5 minutes; 95 ℃, 20 seconds; 55 cycles of 95 ℃ for 3 seconds, 60 ℃ for 30 seconds. Fluorescence was measured at 60 ℃ for each cycle. All assays were performed with at least three technical replicates.

7.1 Detection the HCV RNA in the quantitative reference serum sample by MOF enrichment method

HCV RNA positive quantitative reference sample (Daan genes™, China, DA-Z070), HCV RNA concentration are 1.0E+03, 1.0E+04, 1.0E+05, and 1.0 E+06 IU/mL, respectively). These was used for quantitative detecting the HCV RNA concentration in the clinical plasma. RNA was extracted from 200 μL HCV RNA positive quantitative reference plasma sample, clinical blood from HCV-infected patients and none-HCV-infected individual by our MOF enrichment method, respectively. And these RNAs were used as template to operate the RT-qPCR, and the data of the HCV RNA positive quantitative reference plasma sample was used to calculated the linear relationship between the quantification cycle (Ct) values, and the log hepatitis C virus (HCV) RNA concentration ($Y = 44.2235-2.8955x$, $R^2 = 0.9982$; $Y = 48.417-3.656x$, $R^2 = 0.9905$; $Y = 46.507-3.281x$, $R^2 = 0.9981$, three biological repeats, see the Figure S11). The very good linear working curve also shows that the MOF extraction method has high accuracy and stability.

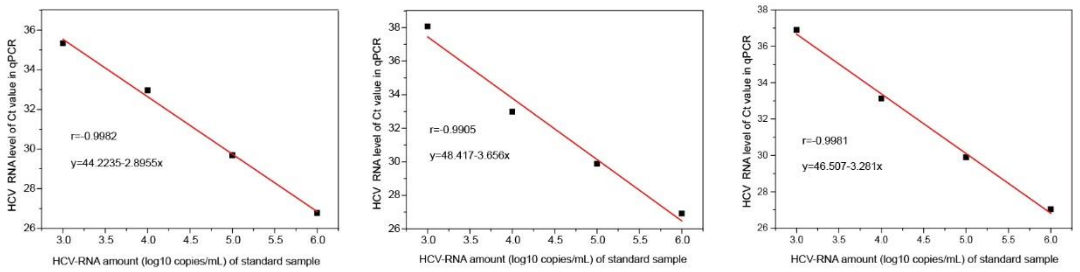

Figure S16. Linear relationship between the quantification cycle (Ct) values, and the log HCV RNA copy number, in three different experiments.

7.2 Quantitative detection HCV RNA through MOF method and comparation with clinical method

The performance of our MOF method by RT-qPCR was compared to that of commercial diagnostic Kit, Quantification of Hepatitis C Virus RNA (Daan genes^{TM}, China, DA-Z070) by analyzing HCV RNA in the clinical serum samples from HCV-infected patients and none-HCV-infected people. The operation steps of the Diagnostic Kit for Quantification of Hepatitis C Virus RNA were performed according to the instructions provided by the manufacturer. This commercial kit contains HCV RNA positive quantitative reference sample (HCV RNA concentration are 1.0E+03, 1.0E+04, 1.0E+05, and 1.0 E+06 IU/mL, respectively), which was also used for quantitively detection in the MOF enrichment method.

The HCV copy number results are shown in Table S2 of the two methods, then we perform the consistency test of the two methods.

Table S2. HCV copy number detected by MOF method and clinical method.

		copys/mL		log_{10} (copys/mL+1)	
	sample number	clinical assay	MOF assay	clinical assay	MOF assay
Negtive	1	<20	3.63E+02	0	2.559354
	2	<20	N/A	0	0
	3	<20	4.58E+02	0	2.660558
	4	<20	N/A	0	0
	5	<20	N/A	0	0

	6	<20	N/A	0	0
	7	<20	N/A	0	0
	8	<20	N/A	0	0
	9	<20	N/A	0	0
	10	<20	N/A	0	0
	11	<20	N/A	0	0
	12	<20	N/A	0	0
	13	53	N/A	1.724276	0
	14	<20	N/A	0	0
	15	<20	N/A	0	0
	16	<20	N/A	0	0
	17	<20	N/A	0	0
	18	<20	N/A	0	0
	19	<20	4.63E+02	0	2.665956
	20	<20	N/A	0	0
Positive	21	2.61E+07	1.00E+07	7.416641	7.001727
	22	6.42E+06	1.61E+05	6.807535	5.205839
	23	2.94E+07	2.66E+06	7.468347	6.42497
	24	6.29E+04	8.99E+04	4.798658	4.953726
	25	1.08E+07	1.11E+07	7.033424	7.046624
	26	4.38E+06	3.93E+06	6.641474	6.594636
	27	9.69E+06	2.58E+06	6.986324	6.411765
	28	2.49E+06	4.41E+05	6.3962	5.644449
	29	2.94E+06	6.34E+05	6.468347	5.802195
	30	6.30E+05	4.31E+05	5.799341	5.634564

Data <20 is recorded as 0, and then calculate $\log_{10}$ (copys/mL+1)

Table S3. Comparison of HCV-RNA detection results between two methods

Clinical method	MOF method		Total
	Positive	Negative	
Positive	10	1	11
Negative	3	16	19
Total	13	17	30

In this study, kappa statistical test was used to analyze the consistency of the two methods to detect HCV virus copy number in 30 specimens, showing that the two

methods had high consistency (Kappa value=0.724, P=0.000063). The results showed that there were 10 positive samples and 17 negative samples for both methods. The clinical test results were negative, and there were 3 samples tested positive by the MOF method. Only 1 sample was a positive sample by the clinical test results, and the MOF method test results were negative (Table S3). It should be noted that the HCV virus copy number of the 4 samples with inconsistent detection is very low, and the judgment of these samples needs further verification. However, in samples with high HCV virus copy number, the positive diagnostic consistency of the two methods was extremely high. We analyzed the correlation of HCV virus copy number between the two methods in 10 samples with both positive diagnoses, and the result were consistent (r = 0.754, P =0.011788), as showed in Fig. S10.

The HCV virus copy number detected by the MOF method in the same sample is highly consistent with the clinical detection method, indicating that the MOF method for extracting HCV virus RNA from blood has high accuracy and good stability. In positive samples, the number of HCV virus copies detected by the MOF method is slightly lower than that of the clinical method, mainly because the clinical method is only a si mple lysate followed by direct qPCR detection, while the MOF method also includes a step of purifying nucleic acid, which will lead to partial loss. The purified nucleic acid is suitable for small-volume enzyme ligation reactions for downstream high-throughput sequencing detection.

8. HBV copy number detection by MOF-sequencing and compared with clinical detecting

Serum HBV DNA, hepatitis B surface antigen (HBsAg) and hepatitis B core-related antigens (HBcrAgs), have been shown to correlate with intrahepatic covalently closed circular DNA (cccDNA), furthermore, serum HBV RNA has been considered as a new biomarker for especially in virally suppressed patients with low detectable HBV DNA under nucleos(t)ide analogues therapy. Guo et al. summarized the reported correlations between serum HBV RNA and other serological markers,

and showed that serum HBV RNA and HBV DNA had a good correlation, and the correlation coefficient, r was around 0.7. The overall correlation coefficient with serum HBsAg was lower than that with HBV DNA (*6*). It is worth mentioning that some patients had undetectable serum HBV DNA in the CHB patient group, but HBV RNA signals could be detected (Fig. 4E), probably due to the difference in extraction and detection methods. Because the clinical method used qPCR detection after serum lysis, while our method used transcriptome sequencing after extraction and purification. It also may be due to inconsistent changes in HBV RNA and DNA levels after drug treatment (*6*).

Table S4. HBV copy number detected by MOF assay and clinical assay.

		MOF assay（NGS）		clinical assay（qPCR）		clinical assay (antigen detection)
	samples	HBV RNA reads per 10 million/0.5mL	$\log_{10}$(HBV RNA reads per 10 million/0.5mL+1)	HBV DNA copies/mL	$\log_{10}$(HBV DNA copies/mL+1)	HBsAg
CHB	1	54.92316543	1.747591746	<20	0	50.04
	2	315.6273499	2.500548426	2550000	6.4065404	-
	3	9.624514022	1.026309074	<20	0	1680
	4	15896.01163	4.201315492	398000000	8.5998831	>52000
	5	39.75627636	1.610194498	49100	4.6910903	-
	6	14.4399849	1.188646871	401000	5.6031455	341.2
	7	116.5940286	2.070385269	164000000	8.2148439	9552
	8	56.49801477	1.75965285	8930	3.9509001	250
	9	16.50957576	1.243275624	<20	0	0
	10	46.03085172	1.672382844	3078	3.4884097	>250
	11	7531.949015	3.876965028	21070000	7.3236646	2553
	12	1.741102877	0.437925335	479	2.6812412	-
	13	3.777730073	0.67922161	<20	0	1517
	14	4.327861786	0.72655295	<20	0	2064
	15	37.32132962	1.583440569	416000	5.6190944	11.15
	16	4.230522586	0.718545082	<20	0	295.4
	17	22.23665752	1.366173657	56	1.7558749	583.5
Normal	18	0	0	-	-	-

	19	0	0	-	-	-
	20	33.09302283	1.532665509	-	-	-
	21	0	0	-	-	-
	22	0	0	-	-	-
	23	0	0	-	-	-
	24	0	0	-	-	-
	25	0	0	-	-	-

Data <20 is recorded as 0, and then calculate $\log_{10}$ (copys/mL+1)

9. Sequencing and data analysis of cfRNA in HCC, CHB and normal group

9.1 The inclusion and exclusion criteria for HCC, CHB, and normal group

HCC patients were confirmed by pathology, without other cancers. The diagnosis of CHB is consistent with the American Association for the Study of Liver Diseases (AASLD) 2018 hepatitis B guidance (*5*). The above two groups of patients were excluded from other viral infections except HBV. Healthy controls were recruited with normal physical examinations, and individuals with severe chronic diseases (e.g., diabetes and hypertension) and/or a family history of cancer through questionnaires were excluded. The characteristics of training and validation population was list in Table S6 and S7.

9.2 Transcriptome-wide characterization of cfRNA in HCC, CHB and normal individuals

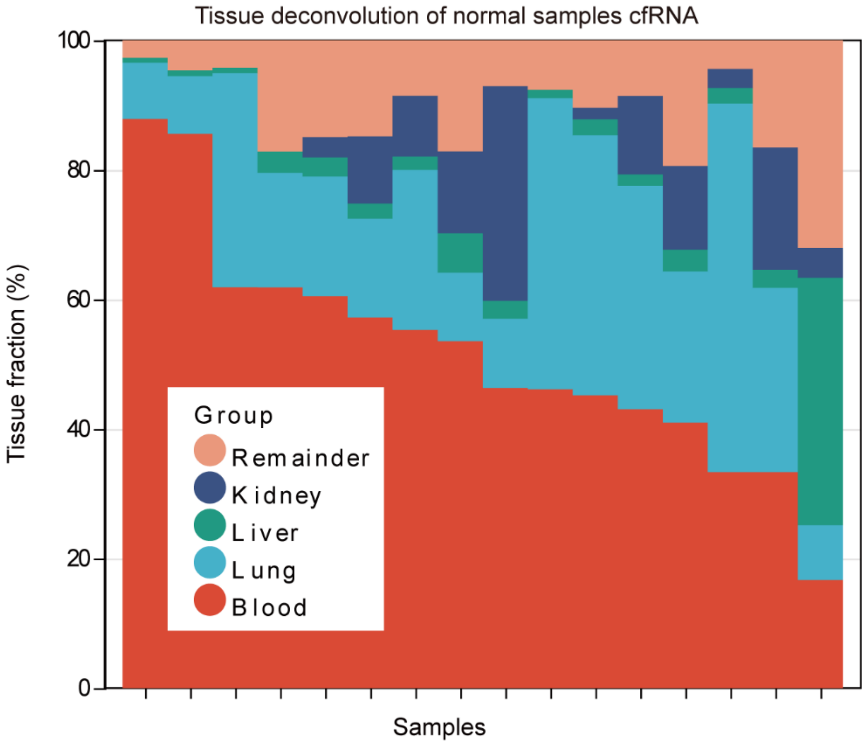

Figure S17. Tissue deconvolution for cfRNA from 16 normal serum samples.

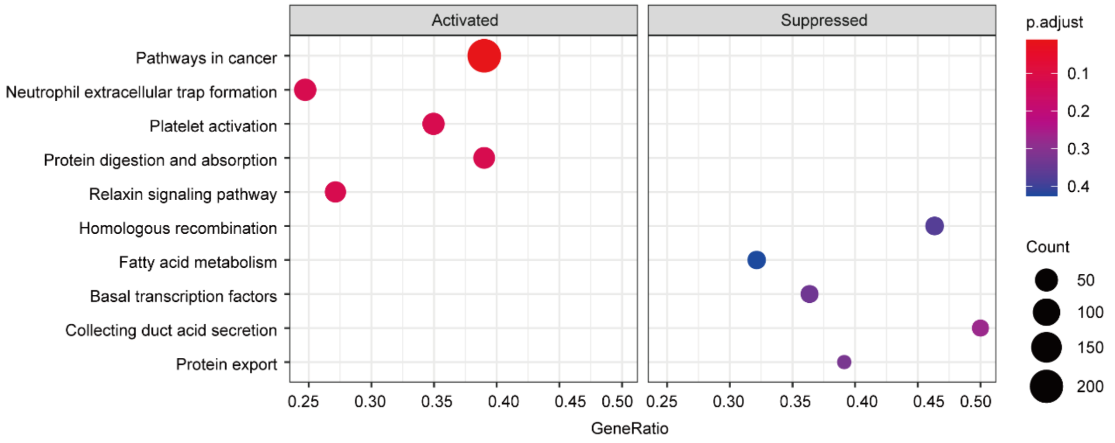

Figure S18. Gene Set Enrichment Analysis (GSEA) for differentially expressed genes between HCC and normal serum samples.

9.3 Identification of HCC-specific cfRNA candidates and evaluation their potential diagnostic value for HCC

A considerable number of cfRNAs were upregulated in HCC samples compared with healthy controls (**Figure S19**). However, these upregulated cfRNAs rarely overlapped with upregulated mRNAs in HCC tissues (**Figure S20**). We believe that

there are two reasons for the low number of overlaps: the first one is that cfRNA represents a mixture of transcripts reflecting the health status of multiple tissues, thereby affording broad clinical utility. However, several aspects about the physiologic origins of cfRNA, including that which cell types are contributors of cfRNA origin and which RNA molecules in cells are released into the bloodstream, remain unknown. (Figure S17). The other reason is that we set |logFC| > 1, we wanted to get cfRNAs with a greater degree of difference. At the same time, we got cfRNAs with a relatively high base expression by setting the base mean value above 10, thus excluding a considerable number of cfRNAs that could potentially overlap with mRNAs in liver cancer tissues. Therefore, upregulated cfRNAs rarely overlapped with upregulated mRNAs in HCC tissues.

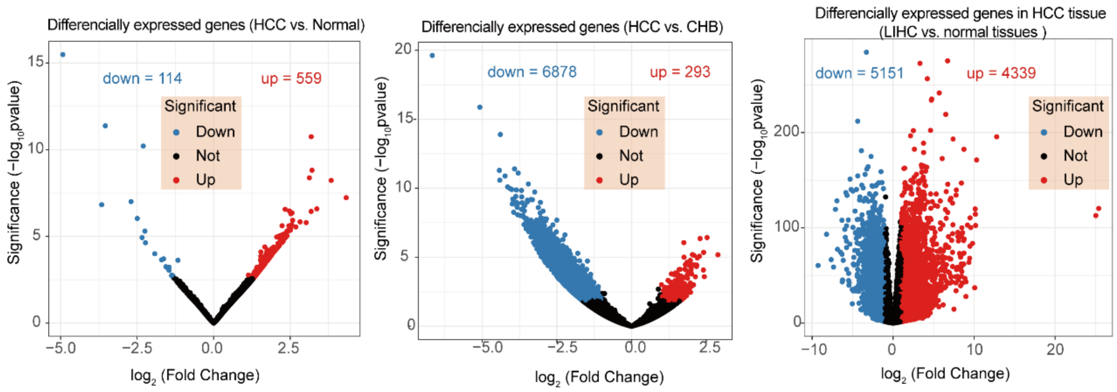

Figure S19. Volcano plot of differentially expressed genes between HCC and normal serum samples, HCC and CHB serum samples in training set, and HCC and non-HCC tissue samples (TARGET GTEx dataset), |logFC| > 1 and P <0.05 was used as the cutoff criteria for the differential expression analysis.

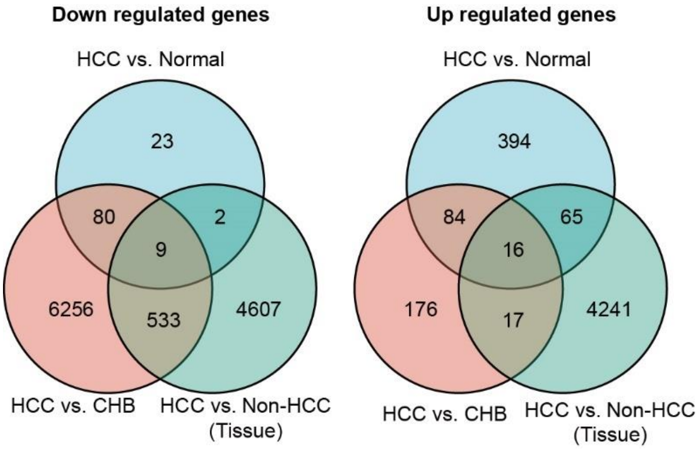

Figure S20. Overlapping features of differentially expressed genes between HCC and normal serum samples in training set, HCC and CHB serum samples in training set, and HCC and non-HCC tissue samples (TARGET GTEx dataset).

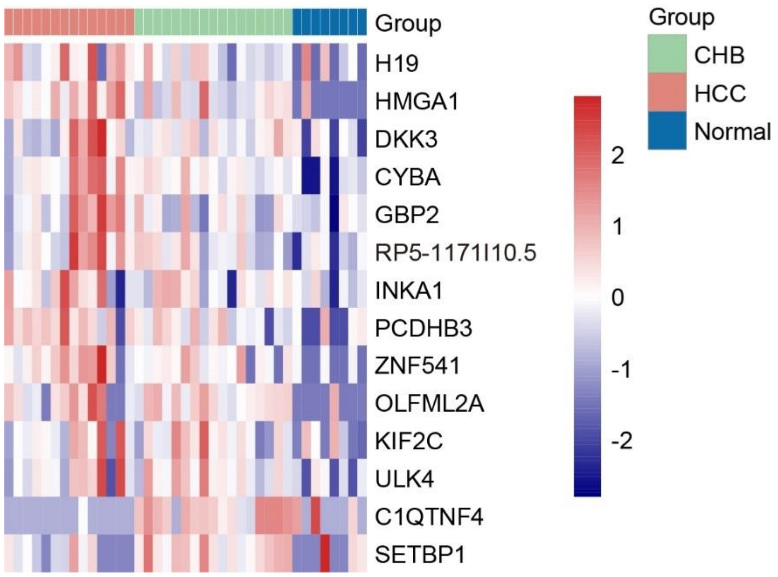

Figure S21. The distribution of the 14 cfRNA candidates expression levels in HCC, CHB and normal group of the training set.

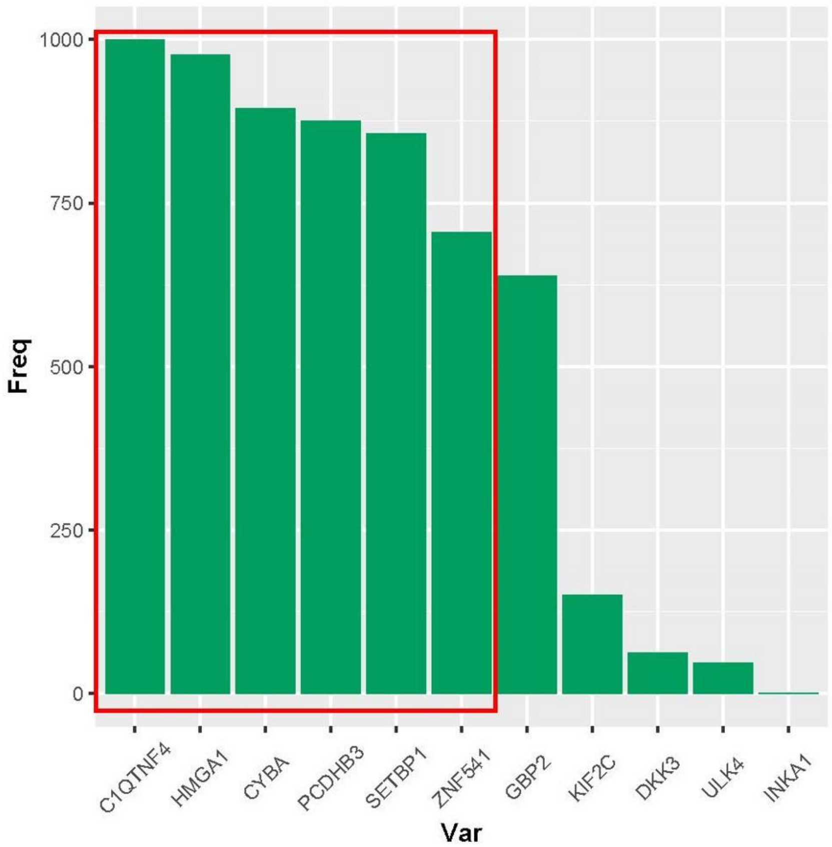

Figure S22. Selection of the cfRNA candidates through lasso regression analysis with 1000 iteration for classifying patients with HCC and non-HCC.

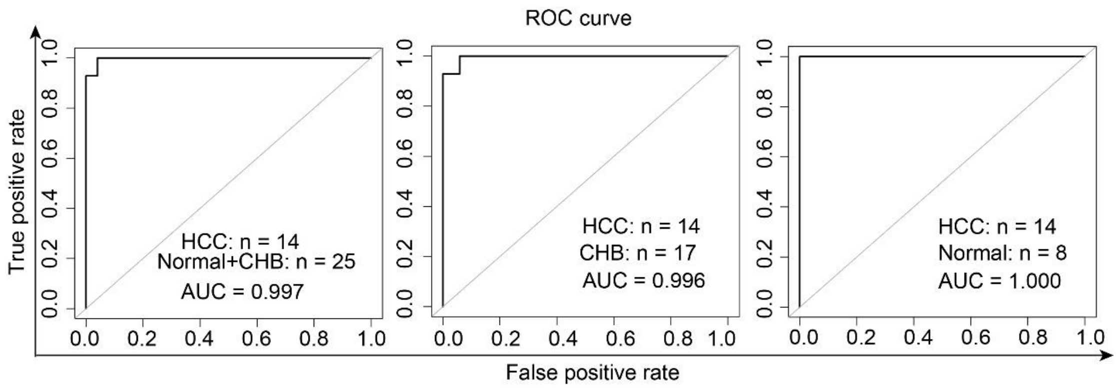

Figure S23. ROC curve of the cfRNA signature for discriminating HCC from CHB and normal together, or from CHB and normal separately in the training set.

1

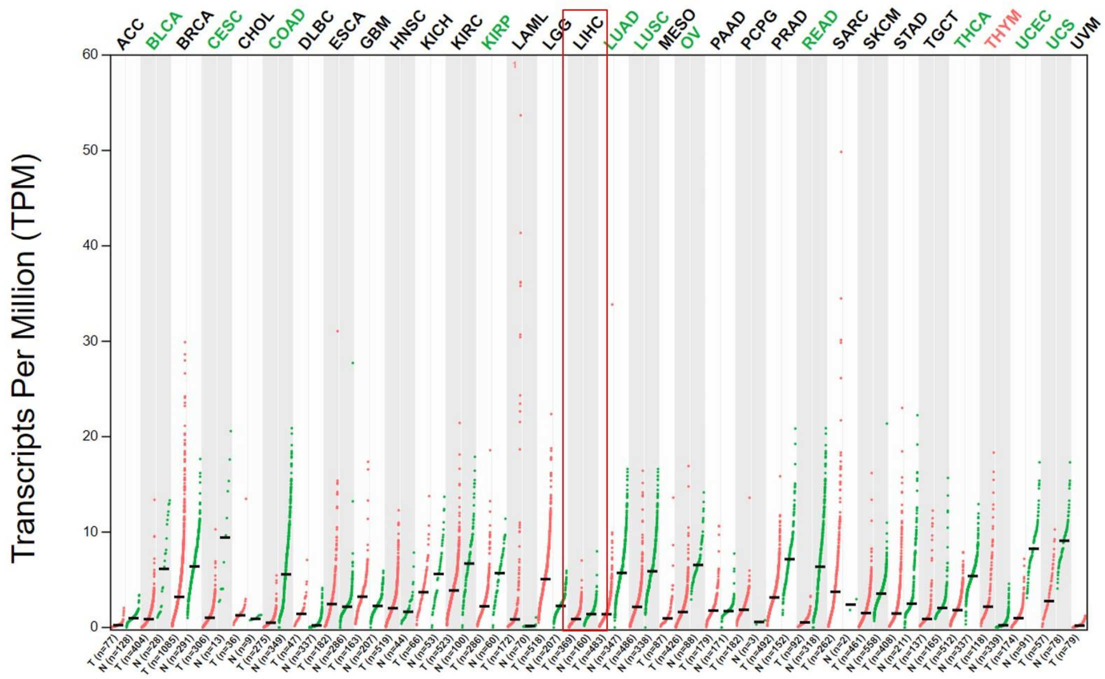

Figure S24. Tissue-wise expression of *SETBP1* gene in different cancer types (Data from database of Gene Expression Profiling Interactive Analysis, http://gepia2.cancer-pku.cn/#analysis)

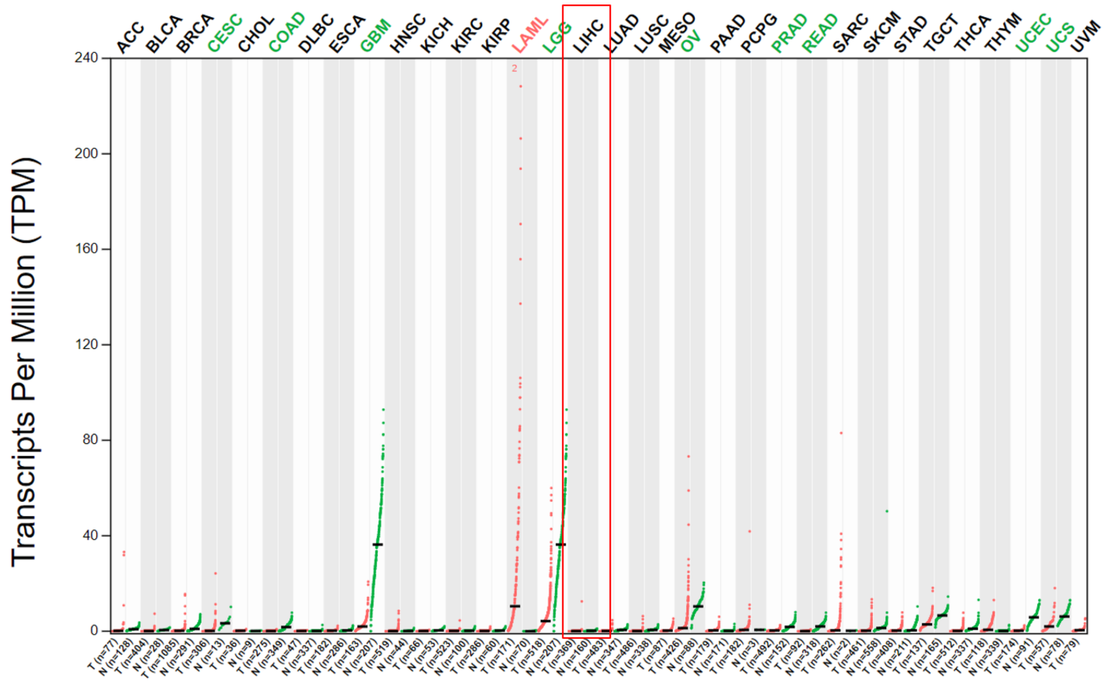

Figure S25. Tissue-wise expression of *C1QTNF4* gene in different cancer types (Data from database of Gene Expression Profiling Interactive Analysis, http://gepia2.cancer-pku.cn/#analysis)

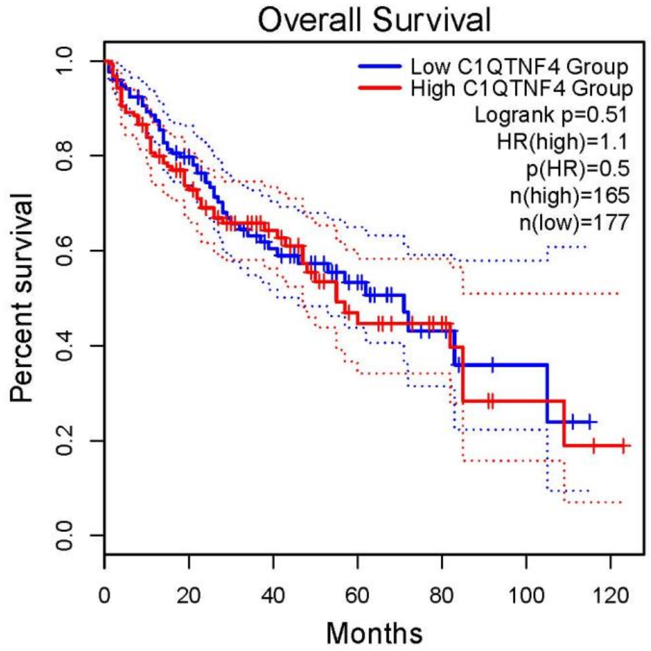

Figure S26. The survival curve of C1QTNF4 in hepatocellular carcinoma tissue (data from Data from database of Gene Expression Profiling Interactive Analysis, http://gepia2.cancer-pku.cn/#survival).

Table S5. Characteristics of training population

**Characteristics**	**HCC patients (n=14)**	**CHB patients (n=17)**	**Healthy controls (n=8)**	***p* value[a]**	***p* value[b]**	***p* value[c]**
Age, years	59.6 (9.6)	40.1 (13.0)	44.4 (11.3)	4.07E-04	4.08E-03	3.36E-01
Males, n (%)	13 (92.9)	12 (70.6)	6 (75.0)	1.85E-01	5.27E-01	1.00E+00
ALT, U/L	52.4 (58.5)	79.2 (160.5)	20.0 (9.9)	3.82E-01	7.91E-02	2.17E-01
AST, U/L	42.5 (33.3)	43.0 (49.7)	20.1 (5.9)	1.35E-01	8.91E-03	9.17E-02
γGT, U/L	110.1 (83.7)	37.4 (42.3)	17.4 (4.5)	5.04E-03	5.18E-04	1.67E-01
ALP, U/L	152.9 (109.7)	84.9 (27.1)	91.0 (19.3)	7.68E-03	9.31E-02	3.51E-01
HBV DNA-positive	4 (28.6)	9 (52.9)	—	4.52E-01	NA	NA
HBsAg-positive	10 (71.4)	14 (82.4)	—	2.03E-01	NA	NA
Total bilirubin, µmol/L	17.4 (7.4)	18.0 (7.6)	17.3 (5.0)	7.83E-01	7.94E-01	8.23E-01
Direct bilirubin, µmol/L	5.3 (2.7)	6.6 (2.0)	3.2 (1.0)	1.47E-01	7.91E-02	7.75E-04
Unconjugated bilirubin, µmol/L	12.0 (5.8)	11.5 (6.0)	14.1 (4.1)	9.27E-01	4.78E-01	7.95E-02
Total proteins, g/L	68.5 (8.0)	74.2 (4.0)	75.3 (3.4)	1.68E-02	1.87E-02	6.82E-01
Albumin, g/L	35.4 (5.9)	44.7 (4.2)	48.4 (3.5)	8.74E-04	1.14E-03	7.92E-02
Globin,g/L	33.6 (7.9)	29.6 (2.8)	26.9 (3.0)	1.03E-01	1.87E-02	9.29E-02
Glucose, mmol/L	6.1 (3.5)	6.0 (2.0)	4.8 (0.3)	6.99E-01	3.83E-01	1.25E-01
AFP, ng/mL	1875.0 (3545.3)	5.7 (8.1)	4.1 (1.8)	3.02E-01	4.41E-01	5.95E-01

Means (standard deviation) for continuous variables and n (%) for categorical variables

Fisher's exact test for categorical variables and Wilcoxon test for continuous variables; [a]Comparison between HCCs and controls; [b]Comparison between HCCs and CHBs; [c]Comparison between CHBs and controls

ALT, alanine aminotransfrase; AST, aspartate transaminase; ALP, alkaline phosphatase; γGT, gamma-glutamyl transpeptidase; AFP, alpha-fetoprotein; NA, not available

Table S6. Characteristics of validation population

Characteristics	HCC patients (n=7)	CHB patients (n=4)	Healthy controls (n=8)	*p* value[a]	*p* value[b]	*p* value[c]
Age, years	63.6 (5.0)	39.8 (11.8)	47.0 (6.1)	1.06E-02	1.75E-03	4.43E-01
Males, n (%)	3 (42.9)	2 (50.0)	4 (50.0)	1.00E+00	1.00E+00	1.00E+00
ALT, U/L	31.7 (9.5)	53.5 (47.7)	21.9 (12.6)	7.48E-01	1.37E-01	1.06E-01
AST, U/L	59.7 (42.1)	41.5 (28.3)	21.3 (4.5)	3.52E-01	7.99E-03	6.04E-02
γGT, U/L	277.3 (299.3)	19.5 (3.9)	20 (NA)	9.52E-03	2.86E-01	1.00E+00
ALP, U/L	309.5 (241.4)	77.8 (14.7)	91.0 (NA)	1.14E-01	8.57E-01	8.00E-01
HBV DNA-positive	2 (28.6)	2 (50.0)	—	1.00E+00	NA	NA
HBsAg-positive	2 (28.6)	3 (75.0)	—	4.29E-01	NA	NA
Total bilirubin, μmol/L	44.7 (28.2)	11.5 (5.0)	15.5 (4.6)	1.91E-02	3.19E-02	3.15E-01
Direct bilirubin, μmol/L	19.2 (15.5)	5.1 (1.9)	3.8 (2.7)	3.81E-02	8.16E-03	7.27E-02
Unconjugated bilirubin, μmol/L	25.5 (16.6)	6.4 (3.2)	12.0 (5.0)	1.91E-02	7.34E-02	7.27E-02
Total proteins, g/L	65.9 (6.3)	75.5 (3.0)	75.8 (NA)	1.91E-02	2.86E-01	1.00E+00
Albumin, g/L	34.3 (7.6)	44.4 (3.1)	42.8 (NA)	3.81E-02	5.71E-01	1.00E+00
Globin,g/L	31.7 (7.7)	31.1 (1.6)	33.0 (NA)	1.00E+00	1.00E+00	4.00E-01
Glucose, mmol/L	5.8 (0.9)	5.3 (0.7)	5.0 (0.6)	5.71E-01	6.53E-02	4.97E-01
AFP, ng/mL	92.4 (153.2)	2.6 (1.7)	3.4 (1.4)	5.17E-01	7.79E-01	3.76E-01

Means (standard deviation) for continuous variables and n(%) for categorical variables

Fisher's exact test for categorical variables and Wilcoxon test for continuous variables; [a]Comparison between HCCs and controls; [b]Comparison between HCCs and CHBs; [c]Comparison between CHBs and controls

ALT, alanine aminotransfrase; AST, aspartate transaminase; ALP, alkaline phosphatase; γGT, gamma-glutamyl transpeptidase; AFP, alpha-fetoprotein; NA, not available

Table S7. Literature survey on the function of 6 genes and their association with liver disease

	Genes	RNA type	Biological process	Molecular function	Number of Pubmed database search entries: search "genes and human"	Number of Pubmed database search entries: search "genes and liver and human	Rough description	Research PMID
down	**C1QTNF4**	protein coding	Transcriptional regulators	Cytokine	11	0	Promotion cell survival in human cancer cells	21658842
	**SETBP1**	protein coding	Transcriptional regulators	DNA-binding	207	0	Prediction non-small cell lung cancer	30224757
up	**HMGA1**	protein coding	Transcription, Transcription regulation	DNA-binding	922	49	Promotion tumor growth and migration in HCC	27855356, 33332531, 29152644, 29511732, 34407055, 32989212, 14714251
	**PCDHB3**	protein coding	Cell adhesion	—	3	0	Related with colorectal cancer and ovarian cancer	25032869
	**CYBA**	protein coding	Electron transport, Transport	Oxidoreductase	792	25	Related to hepatitis and liver fibrosis	30087027, 31094169, 20660993, 27740521, 24864467, 31969899, 25888935
	**ZNF541**	protein coding	Differentiation, Transcription regulation	Developmental protein, Repressor	2	0	—	—

Table S8. Sequences of DNA/RNA used in this study

Experiment	Name	Sequence (from 5' end to 3' end)	Modification
cfDNA qPCR	ALU115- F	CCTGAGGTCAGGAGTTCGAG	
	ALU115- R	CCCGAGTAGCTGGGATTACA	
HCV RT-qPCR	HCV taqman probe	CCGGGGCACTCGCAAGCACCC	5'6-FAM, 3'MGB
	HCV forward primer	TGCACGGTCTACGAGAC	
	HCV reverse primer	GCCTTGTGGTACTGCCTGAT	
Polyacrylamide gel electrophoresis	FAM-33nt-ssDNA	FAM-CCGCGGCCAGGCT AGCTACAACGACCTGGACGA	FAM
	FAM-22nt-ssDNA	FAM-TAACCAATGTGCAGACTACTGT	FAM
	FAM-22nt-ssRNA	FAM-rCrGrGrGrUrArGrArGrA rGrGrGrCrArGrUrGrGrGrArG	FAM

Assessment the interference of age (and gender) on the predictive ability of these markers and model score for HCC

Age may be a confounding factor in the diagnosis of HCC by the model in this work, and therefore the interference of age (and gender) on the predictive ability of these markers and model score for HCC needs to be critically assessed.

First, we assessed the correlation of the six markers and the model score with age, and found that only C1QTNF4 and the model score were moderately correlated with age as well as statistically significant. The other markers were not statistically significant despite some weak correlation with age (Figure S24). From the model equation cfRNA-score = (-0.65310) *C1QTNF4 + 0.15879*CYBA + 0.27373*HMGA1 + 0.29307*PCDHB3 + (-0.21822) *SETBP1 + 0.12482*ZNF541 +

(-2.25578), score is mainly contributed by negative C1QTNF4 expressions (with the largest absolute values of the coefficients) and they show a negative correlation with age. Therefore, assessing the effect of age on the predictive ability of the model Score depends mainly on the effect of age on the predictive ability of C1QTNF4. Further, we used multifactorial logistic regression to assess the interference of age/sex on the predictive ability of markers and model score for HCC (Table S6). We found that without correcting for any factors (model 1), age, CYBA, HMGA1, PCDHB3, and ZNF541 were risk factors for HCC, and C1QTNF4 and SETBP1 were protective factors for HCC. Corrected for age (model 2) or corrected for age and sex (model 3), the odds ratios (ORs) of 6 markers and score were only mildly altered. Probably due to the small sample size, CYBA and SETBP1 did not reach statistical significance after complete correction (model 3). Notably, C1QTNF4 is still shown to be independent protective factors for HCC after correcting for age (model 2) or correcting for age and sex (model 3). In summary, our model was limitedly disturbed by age differences and demonstrated an independent predictive value for hepatocellular carcinoma.

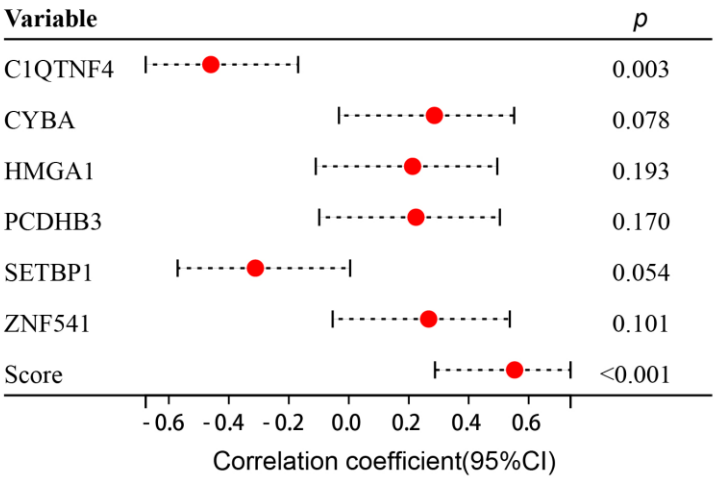

Figure S27. Pearson's correlation between markers and age.

Table S9. The odds ratios (ORs) of 6 markers and score

Variable	Model 1		Model 2 (adjust age)		Model 3 (adjust age/ gender)	
	**OR (95% Cl)**	***p***	**OR (95% Cl)**	***p***	**OR (95% Cl)**	***p***
Age	1.14 (1.06-1.25)	0.002	--	--	--	--
Gender	0.20 (0.01-1.31)	0.150	0.03 (0-0.53)	0.040	--	--
C1QTNF4	0.20 (0.03-0.5)	0.014	0.24 (0.04-0.59)	0.019	0.25 (0.04-0.65)	0.028
CYBA	1.89 (1.18-3.76)	0.026	2.29 (1.12-7.08)	0.069	2.04 (1.04-5.85)	0.091
HMGA1	1.79 (1.23-2.96)	0.008	1.83 (1.14-3.52)	0.028	2.06 (1.19-4.62)	0.029
PCDHB3	2.17 (1.17-5.13)	0.035	2.05 (1.01-5.38)	0.080	2.83 (1.19-9.76)	0.045
SETBP1	0.59 (0.34-0.94)	0.040	0.65 (0.32-1.19)	0.183	0.77 (0.35-1.64)	0.481
ZNF541	2.08 (1.2-4.47)	0.025	2.21 (1.06-6.12)	0.067	2.91 (1.17-10.37)	0.049
Score	0.196		0.999			

Reference

1. N. Umetani, *et al.*, Prediction of Breast Tumor Progression by Integrity of Free Circulating DNA in Serum. *J. Clin. Oncol.* **24**, 4270-4276 (2006).
2. M. Martin, Cutadapt removes adapter sequences from high-throughput sequencing reads. *EMBnet J.* **17**, 10-12 (2011).
3. A. Dobin, *et al.*, STAR: ultrafast universal RNA-seq aligner. *Bioinformatics (Oxford, England)* **29**, 15-21 (2013).
4. L. Chen, *et al.*, Hepatitis C Virus RNA Real-Time Quantitative RT-PCR Method Based on a New Primer Design Strategy. *J. Mol. Diagn.* **18**, 84-91 (2016).
5. N. A. Terrault, *et al.*, Update on prevention, diagnosis, and treatment of chronic hepatitis B: AASLD 2018 hepatitis B guidance. *Hepatology* **67**, 1560-1599 (2018).
6. S. Liu, B. Zhou, J. D. Valdes, J. Sun, H. Guo, Serum Hepatitis B Virus RNA: A New Potential Biomarker for Chronic Hepatitis B Virus Infection. *Hepatology* **69**, 1816-1827 (2019).
7. M. H. Larson, *et al.*, A comprehensive characterization of the cell-free transcriptome reveals tissue- and subtype-specific biomarkers for cancer detection. *Nat. Commun.* **12**, 2357 (2021).
